# Supplementary material for: The SIESTA (SEAAV Integrated evaluation sedation tool for anaesthesia) project: Initial development of a multifactorial sedation assessment tool for dogs
Source: PLoS One. 2020 Apr 1;15(4):e0230799. doi: 10.1371/journal.pone.0230799 (PMC7112187; doi:10.1371/journal.pone.0230799)
Supplement: S1 Appendix — Final version of the questionnaire distributed to the collaborators. (PDF) [file pone.0230799.s001.pdf]

ESCALA MULTIFACTORIAL DE SEDACIÓN

v1.0

FECHA	EVALUADOR/CLÍNICA

RAZA	MOTIVO
SEXO H ◉ M ○	ASA I ◉ II ○ III ○ IV ○ V ○ E ☐
EDAD	PESO

PROTOCOLO		DOSIS	VÍA	PROTOCOLO		DOSIS	VÍA
DEX	☐		IV ○ IM ○ SC ○	BUT	☐		IV ○ IM ○ SC ○
MED	☐		IV ○ IM ○ SC ○	MRF	☐		IV ○ IM ○ SC ○
ACP	☐		IV ○ IM ○ SC ○	MTD	☐		IV ○ IM ○ SC ○
MDZ	☐		IV ○ IM ○ SC ○	PET	☐		IV ○ IM ○ SC ○
DZP	☐		IV ○ IM ○ SC ○	FEN	☐		IV ○ IM ○ SC ○
ALX	☐		IV ○ IM ○ SC ○	BUP	☐		IV ○ IM ○ SC ○
KET	☐		IV ○ IM ○ SC ○	AINES	☐		IV ○ IM ○ SC ○
OTROS 1	☐		IV ○ IM ○ SC ○	OTROS 2	☐		IV ○ IM ○ SC ○

ALERTA	Sí ◉ No ○
ESTADO MENTAL	Calmado/tranquilo ◉<br>Somnoliento/Obnubilado ○<br>Inconsciente/Dormido ○<br>Nervioso/Agitado/Inquieto ○<br>Disfórico/Delira ○<br>Miedoso ○
POSICIÓN CABEZA	Elevada ◉<br>Baja/caída ○<br>Apoyada en mesa/ suelo ○
CAMBIO DE POSTURA	Sin estímulo ◉ Con estímulo ○<br>No cambia ○

COMPORTAMIENTO	Agresivo ☐<br>De huida ☐<br>Autolesivo ☐<br>Defensivo ☐<br>Exploratorio ☐<br>Interactivo ☐
POSTURA	De pie ◉<br>Sentado ○<br>Esternal ○<br>Lateral ○
REFLEJO POSTURAL	Sí ◉ No ○

ATAXIA	Sí ○	No ◉
HIPERACTIVO/HIPERCINÉTICO	Sí ○	No ◉
MOVS EXAGERADOS	Sí ○	No ◉
SIGUE CON LA MIRADA	Sí ○	No ◉
MIRA GIRANDO LA CABEZA	Sí ○	No ◉
TONO MUSCULAR	Normal ◉<br>Relajado ○<br>Tenso ○	

MOVIMIENTOS	Voluntarios ☐<br>Tras un estímulo ☐<br>Con oposición ☐<br>No posibles ☐<br>Bruscos/rápidos ☐
MOVIMIENTOS	Normales ◉<br>Aumentados ○<br>Disminuidos ○<br>Ausentes/nulos ○

POSICIÓN DEL OJO	Centrado ◉<br>Rotado ○	
PÁRPADOS	Abiertos ◉<br>Cerrados ○<br>Excesivamente abiertos ○	
REFLEJO PALPEBRAL	Sí ◉	No ○
3er PÁRPADO VISIBLE	Sí ○	No ◉
NISTAGMO	Sí ○	No ◉
MIDRIASIS	Sí ○	No ◉

VOCALIZACIÓN	No ◉<br>Ladra ○<br>Lloriquea ○<br>Se queja ○<br>Aúlla ○	
SIALORREA	Sí ○	No ◉
FR (resp/min)	Normal ◉<br>Aumentada ○<br>Disminuida ○	
JADEO	Sí ○	No ◉

REFLEJO DE AMENAZA	Aumentado ○<br>Normal ◉<br>Disminuido ○<br>Nulo ○
RESPUESTA A LLAMADA	Aumentada ○<br>Normal ◉<br>Disminuida ○<br>Nula ○
RESPUESTA A PALMADA	Aumentada ○<br>Normal ◉<br>Disminuida ○<br>Nula ○

POSIBLE COGER EXT. ANTERIOR	Sí ◉	No ○
RESPUESTA A PRESIONAR PULPEJO EXT. POSTERIOR	Aumentado ○<br>Normal ◉<br>Disminuido ○<br>Nulo ○	
RESPUESTA A LA SUJECIÓN	No es posible sujetarlo ○<br>Se resiste y sólo es posible con fuerza bruta ○<br>Se opone, posible con sujeción firme ◉<br>Se deja, pero es necesario cierta sujeción para mantener la postura ○<br>Permanece en la postura deseada sin sujeción ○	
NECESITA BOZAL	Sí ○	No ◉

PROCEDIMIENTO FINAL/MOTIVO DE LA SEDACIÓN	Sí, fácilmente ○<br>Sí, con dificultad ○<br>No posible ◉		
RASURADO	Sí ○	No ○	N/A ◉
VENOCLISIS	Sí ○	No ○	N/A ◉

CATÉTER VENOSO	Sí ○	No ○	N/A ◉
CATÉTER ARTERIAL	Sí ○	No ○	N/A ◉
CATÉTER CENTRAL	Sí ○	No ○	N/A ◉
RADIOGRAFÍA	Sí ○	No ○	N/A ◉
CAMPO QUIRÚRGICO	Sí ○	No ○	N/A ◉

GRADO SEDACIÓN No sedación ◉ Ligera ○ Moderada ○ Profunda ○ Excitación ○

OBSERVACIONES

RESET **ENVIAR**
