# Supplementary material for: The SIESTA (SEAAV Integrated evaluation sedation tool for anaesthesia) project: Initial development of a multifactorial sedation assessment tool for dogs
Source: PLoS One. 2020 Apr 1;15(4):e0230799. doi: 10.1371/journal.pone.0230799 (PMC7112187; doi:10.1371/journal.pone.0230799)
Supplement: S1 Data — (PDF) [file pone.0230799.s002.pdf]

Degree	Alertness	Head Elevation	Posture	Demenour	Ataxia
GRADO	ALERTA	CABEZA	POSTURA	MENTAL	ATAXIA
DESPIERTO	YES	ELEVADA	PIE	TRANQUILO	NO
DESPIERTO	YES	BAJA	PIE	DORMIDO	NO
DESPIERTO	YES	BAJA	ESTERNAL	TRANQUILO	NO
DESPIERTO	YES	ELEVADA	ESTERNAL	TRANQUILO	NO
DESPIERTO	YES	ELEVADA	PIE	TRANQUILO	NO
DESPIERTO	YES	ELEVADA	PIE	TRANQUILO	NO
DESPIERTO	YES	ELEVADA	SENTADO	NERVIOSO	NO
DESPIERTO	YES	ELEVADA	PIE	TRANQUILO	NO
DESPIERTO	YES	ELEVADA	ESTERNAL	NERVIOSO	NO
DESPIERTO	YES	ELEVADA	PIE	MIEDOSO	NO
DESPIERTO	YES	ELEVADA	PIE	TRANQUILO	NO
DESPIERTO	YES	ELEVADA	PIE	NERVIOSO	NO
DESPIERTO	YES	BAJA	PIE	TRANQUILO	NO
DESPIERTO	YES	ELEVADA	PIE	TRANQUILO	NO
DESPIERTO	YES	ELEVADA	PIE	NERVIOSO	NO
DESPIERTO	YES	ELEVADA	ESTERNAL	TRANQUILO	YES
DESPIERTO	YES	ELEVADA	PIE	TRANQUILO	NO
DESPIERTO	YES	ELEVADA	PIE	TRANQUILO	NO
DESPIERTO	YES	ELEVADA	PIE	TRANQUILO	NO
DESPIERTO	YES	ELEVADA	ESTERNAL	TRANQUILO	NO
DESPIERTO	YES	ELEVADA	PIE	TRANQUILO	NO
DESPIERTO	YES	ELEVADA	SENTADO	TRANQUILO	NO
DESPIERTO	YES	ELEVADA	SENTADO	NERVIOSO	NO
DESPIERTO	YES	ELEVADA	PIE	TRANQUILO	NO
DESPIERTO	YES	ELEVADA	PIE	TRANQUILO	NO
DESPIERTO	YES	ELEVADA	PIE	NERVIOSO	NO
DESPIERTO	YES	ELEVADA	SENTADO	NERVIOSO	NO
DESPIERTO	YES	ELEVADA	PIE	NERVIOSO	NO
DESPIERTO	YES	ELEVADA	SENTADO	TRANQUILO	NO
DESPIERTO	YES	ELEVADA	ESTERNAL	NERVIOSO	NO
DESPIERTO	YES	ELEVADA	PIE	TRANQUILO	NO
DESPIERTO	YES	ELEVADA	PIE	NERVIOSO	NO
DESPIERTO	YES	ELEVADA	PIE	MIEDOSO	NO
DESPIERTO	YES	ELEVADA	PIE	NERVIOSO	NO
DESPIERTO	YES	ELEVADA	PIE	TRANQUILO	NO
DESPIERTO	YES	ELEVADA	PIE	NERVIOSO	NO
DESPIERTO	YES	ELEVADA	PIE	TRANQUILO	NO
DESPIERTO	YES	ELEVADA	PIE	NERVIOSO	NO
DESPIERTO	YES	ELEVADA	PIE	NERVIOSO	NO
DESPIERTO	YES	ELEVADA	SENTADO	NERVIOSO	NO
DESPIERTO	YES	ELEVADA	PIE	NERVIOSO	NO
DESPIERTO	YES	ELEVADA	PIE	NERVIOSO	NO
DESPIERTO	YES	ELEVADA	SENTADO	NERVIOSO	NO
DESPIERTO	YES	ELEVADA	PIE	MIEDOSO	NO
DESPIERTO	YES	ELEVADA	PIE	NERVIOSO	NO
DESPIERTO	YES	ELEVADA	PIE	NERVIOSO	NO
DESPIERTO	YES	ELEVADA	PIE	NERVIOSO	NO
DESPIERTO	YES	ELEVADA	PIE	TRANQUILO	NO
DESPIERTO	YES	ELEVADA	PIE	NERVIOSO	NO
DESPIERTO	YES	ELEVADA	PIE	MIEDOSO	NO
DESPIERTO	YES	ELEVADA	PIE	MIEDOSO	NO

DESPIERTO	YES	ELEVADA	PIE	NERVIOSO	NO
DESPIERTO	YES	ELEVADA	PIE	MIEDOSO	NO
DESPIERTO	YES	ELEVADA	PIE	TRANQUILO	NO
DESPIERTO	YES	ELEVADA	PIE	TRANQUILO	NO
DESPIERTO	YES	ELEVADA	PIE	TRANQUILO	NO
DESPIERTO	YES	ELEVADA	ESTERNAL	TRANQUILO	NO
DESPIERTO	YES	ELEVADA	PIE	MIEDOSO	NO
DESPIERTO	YES	ELEVADA	PIE	NERVIOSO	NO
DESPIERTO	YES	ELEVADA	PIE	NERVIOSO	NO
DESPIERTO	YES	ELEVADA	SENTADO	TRANQUILO	NO
DESPIERTO	YES	ELEVADA	ESTERNAL	TRANQUILO	NO
DESPIERTO	YES	ELEVADA	SENTADO	NERVIOSO	NO
DESPIERTO	YES	ELEVADA	PIE	TRANQUILO	NO
DESPIERTO	YES	ELEVADA	PIE	NERVIOSO	NO
DESPIERTO	YES	ELEVADA	PIE	MIEDOSO	NO
DESPIERTO	YES	ELEVADA	PIE	TRANQUILO	NO
DESPIERTO	YES	ELEVADA	PIE	NERVIOSO	NO
DESPIERTO	YES	ELEVADA	PIE	TRANQUILO	NO
DESPIERTO	YES	ELEVADA	PIE	NERVIOSO	NO
DESPIERTO	YES	ELEVADA	PIE	NERVIOSO	NO
DESPIERTO	YES	ELEVADA	PIE	NERVIOSO	NO
DESPIERTO	YES	ELEVADA	PIE	NERVIOSO	NO
DESPIERTO	YES	ELEVADA	PIE	NERVIOSO	NO
DESPIERTO	YES	ELEVADA	PIE	NERVIOSO	NO
DESPIERTO	YES	ELEVADA	PIE	TRANQUILO	NO
DESPIERTO	YES	ELEVADA	PIE	TRANQUILO	NO
DESPIERTO	YES	ELEVADA	PIE	NERVIOSO	NO
DESPIERTO	YES	ELEVADA	PIE	NERVIOSO	NO
DESPIERTO	YES	ELEVADA	PIE	TRANQUILO	NO
DESPIERTO	YES	ELEVADA	PIE	TRANQUILO	NO
DESPIERTO	YES	ELEVADA	ESTERNAL	TRANQUILO	NO
DESPIERTO	YES	ELEVADA	PIE	NERVIOSO	NO
DESPIERTO	YES	ELEVADA	PIE	NERVIOSO	NO
DESPIERTO	YES	ELEVADA	PIE	NERVIOSO	NO
DESPIERTO	YES	ELEVADA	PIE	NERVIOSO	NO
DESPIERTO	YES	ELEVADA	PIE	NERVIOSO	NO
DESPIERTO	YES	ELEVADA	PIE	NERVIOSO	NO
DESPIERTO	YES	ELEVADA	PIE	NERVIOSO	NO
DESPIERTO	YES	ELEVADA	PIE	NERVIOSO	NO
DESPIERTO	YES	ELEVADA	PIE	NERVIOSO	NO
DESPIERTO	YES	ELEVADA	PIE	NERVIOSO	NO
LIGERA	YES	ELEVADA	ESTERNAL	TRANQUILO	NO
LIGERA	YES	BAJA	ESTERNAL	TRANQUILO	YES
LIGERA	YES	BAJA	PIE	TRANQUILO	NO
LIGERA	YES	BAJA	PIE	TRANQUILO	NO
LIGERA	YES	BAJA	ESTERNAL	TRANQUILO	NO
LIGERA	YES	BAJA	ESTERNAL	TRANQUILO	YES
LIGERA	YES	ELEVADA	ESTERNAL	TRANQUILO	NO
LIGERA	YES	BAJA	PIE	TRANQUILO	YES
LIGERA	YES	ELEVADA	SENTADO	TRANQUILO	NO
LIGERA	YES	ELEVADA	ESTERNAL	TRANQUILO	NO
LIGERA	YES	BAJA	SENTADO	TRANQUILO	NO
LIGERA	YES	ELEVADA	SENTADO	TRANQUILO	NO

LIGERA	YES	ELEVADA	ESTERNAL	SOMNOLIENTO	NO
LIGERA	NO	BAJA	SENTADO	SOMNOLIENTO	NO
LIGERA	YES	BAJA	ESTERNAL	TRANQUILO	YES
LIGERA	NO	BAJA	ESTERNAL	TRANQUILO	NO
LIGERA	YES	ELEVADA	SENTADO	TRANQUILO	YES
LIGERA	YES	ELEVADA	PIE	TRANQUILO	NO
LIGERA	YES	BAJA	ESTERNAL	SOMNOLIENTO	YES
LIGERA	YES	ELEVADA	PIE	TRANQUILO	YES
LIGERA	YES	BAJA	ESTERNAL	SOMNOLIENTO	YES
LIGERA	NO	BAJA	LATERAL	TRANQUILO	NO
LIGERA	YES	BAJA	SENTADO	TRANQUILO	NO
LIGERA	YES	BAJA	PIE	TRANQUILO	NO
LIGERA	YES	BAJA	PIE	TRANQUILO	NO
LIGERA	YES	BAJA	PIE	TRANQUILO	NO
MODERADA	YES	SUELO	ESTERNAL	SOMNOLIENTO	NO
MODERADA	NO	BAJA	ESTERNAL	DORMIDO	NO
MODERADA	NO	BAJA	ESTERNAL	DORMIDO	NO
MODERADA	NO	SUELO	ESTERNAL	SOMNOLIENTO	YES
MODERADA	YES	BAJA	ESTERNAL	TRANQUILO	NO
MODERADA	NO	SUELO	ESTERNAL	TRANQUILO	NO
MODERADA	YES	BAJA	ESTERNAL	SOMNOLIENTO	YES
MODERADA	NO	BAJA	ESTERNAL	SOMNOLIENTO	NO
MODERADA	YES	ELEVADA	ESTERNAL	SOMNOLIENTO	NO
MODERADA	NO	SUELO	LATERAL	SOMNOLIENTO	YES
MODERADA	NO	SUELO	ESTERNAL	DORMIDO	NO
MODERADA	YES	BAJA	ESTERNAL	SOMNOLIENTO	NO
MODERADA	NO	SUELO	ESTERNAL	SOMNOLIENTO	NO
MODERADA	NO	SUELO	ESTERNAL	TRANQUILO	YES
MODERADA	YES	SUELO	ESTERNAL	SOMNOLIENTO	YES
MODERADA	YES	SUELO	ESTERNAL	SOMNOLIENTO	YES
MODERADA	NO	SUELO	LATERAL	TRANQUILO	NO
MODERADA	NO	SUELO	LATERAL	DORMIDO	YES
MODERADA	NO	BAJA	ESTERNAL	SOMNOLIENTO	YES
MODERADA	YES	ELEVADA	ESTERNAL	TRANQUILO	YES
MODERADA	NO	BAJA	ESTERNAL	TRANQUILO	NO
MODERADA	NO	BAJA	ESTERNAL	SOMNOLIENTO	NO
MODERADA	YES	BAJA	ESTERNAL	SOMNOLIENTO	NO
MODERADA	YES	BAJA	ESTERNAL	TRANQUILO	NO
MODERADA	NO	SUELO	LATERAL	SOMNOLIENTO	YES
MODERADA	NO	SUELO	ESTERNAL	TRANQUILO	NO
MODERADA	YES	BAJA	ESTERNAL	TRANQUILO	NO
MODERADA	YES	SUELO	LATERAL	TRANQUILO	NO
MODERADA	NO	SUELO	ESTERNAL	TRANQUILO	YES
MODERADA	NO	SUELO	ESTERNAL	TRANQUILO	YES
MODERADA	NO	BAJA	ESTERNAL	SOMNOLIENTO	NO
MODERADA	NO	BAJA	ESTERNAL	TRANQUILO	YES
MODERADA	YES	SUELO	ESTERNAL	SOMNOLIENTO	NO
MODERADA	YES	SUELO	ESTERNAL	SOMNOLIENTO	YES
MODERADA	NO	BAJA	ESTERNAL	TRANQUILO	YES
MODERADA	YES	SUELO	ESTERNAL	TRANQUILO	NO
PROFUNDA	NO	SUELO	ESTERNAL	DORMIDO	NO
PROFUNDA	NO	ELEVADA	LATERAL	DORMIDO	YES
PROFUNDA	NO	SUELO	ESTERNAL	SOMNOLIENTO	YES

PROFUNDA	NO	SUELO	LATERAL	DORMIDO	NO
PROFUNDA	YES	SUELO	ESTERNAL	DORMIDO	YES
PROFUNDA	NO	BAJA	ESTERNAL	SOMNOLIENTO	YES
PROFUNDA	NO	SUELO	LATERAL	SOMNOLIENTO	NO
PROFUNDA	NO	SUELO	LATERAL	TRANQUILO	NO
PROFUNDA	NO	BAJA	ESTERNAL	SOMNOLIENTO	YES
PROFUNDA	NO	SUELO	LATERAL	DORMIDO	YES
PROFUNDA	NO	SUELO	LATERAL	SOMNOLIENTO	YES
PROFUNDA	NO	SUELO	ESTERNAL	DORMIDO	NO
PROFUNDA	NO	SUELO	ESTERNAL	SOMNOLIENTO	NO
PROFUNDA	YES	SUELO	LATERAL	DORMIDO	YES
PROFUNDA	NO	SUELO	ESTERNAL	SOMNOLIENTO	YES
PROFUNDA	NO	SUELO	LATERAL	SOMNOLIENTO	NO
PROFUNDA	YES	SUELO	LATERAL	DORMIDO	NO
PROFUNDA	NO	SUELO	LATERAL	DORMIDO	NO
PROFUNDA	YES	BAJA	ESTERNAL	TRANQUILO	NO
PROFUNDA	NO	SUELO	ESTERNAL	DORMIDO	YES
PROFUNDA	NO	SUELO	LATERAL	DORMIDO	YES
PROFUNDA	YES	BAJA	LATERAL	TRANQUILO	NO
PROFUNDA	NO	SUELO	LATERAL	DORMIDO	NO
PROFUNDA	NO	SUELO	LATERAL	TRANQUILO	YES
PROFUNDA	NO	SUELO	ESTERNAL	SOMNOLIENTO	NO
PROFUNDA	YES	BAJA	LATERAL	TRANQUILO	YES
PROFUNDA	NO	SUELO	LATERAL	SOMNOLIENTO	NO
PROFUNDA	NO	BAJA	LATERAL	DORMIDO	NO
PROFUNDA	NO	SUELO	ESTERNAL	SOMNOLIENTO	YES
PROFUNDA	YES	SUELO	LATERAL	SOMNOLIENTO	YES
PROFUNDA	NO	SUELO	ESTERNAL	SOMNOLIENTO	YES
MODERADA	YES	ELEVADA	SENTADO	TRANQUILO	NO
PROFUNDA	NO	SUELO	LATERAL	DORMIDO	YES
PROFUNDA	NO	BAJA	LATERAL	DORMIDO	NO
PROFUNDA	NO	SUELO	LATERAL	DORMIDO	YES
PROFUNDA	NO	SUELO	ESTERNAL	DORMIDO	NO
PROFUNDA	YES	SUELO	LATERAL	DORMIDO	NO
PROFUNDA	NO	SUELO	LATERAL	SOMNOLIENTO	NO
PROFUNDA	NO	SUELO	ESTERNAL	NERVIOSO	YES
PROFUNDA	YES	SUELO	ESTERNAL	TRANQUILO	YES
PROFUNDA	NO	SUELO	LATERAL	SOMNOLIENTO	YES
PROFUNDA	NO	SUELO	LATERAL	SOMNOLIENTO	YES
PROFUNDA	NO	SUELO	LATERAL	DORMIDO	NO
PROFUNDA	NO	SUELO	LATERAL	SOMNOLIENTO	YES
PROFUNDA	NO	SUELO	LATERAL	DORMIDO	NO
PROFUNDA	NO	SUELO	LATERAL	SOMNOLIENTO	NO
PROFUNDA	NO	SUELO	ESTERNAL	SOMNOLIENTO	YES
PROFUNDA	YES	SUELO	ESTERNAL	SOMNOLIENTO	YES
PROFUNDA	NO	SUELO	LATERAL	SOMNOLIENTO	NO
PROFUNDA	YES	SUELO	ESTERNAL	SOMNOLIENTO	YES
PROFUNDA	NO	SUELO	LATERAL	SOMNOLIENTO	YES

Hyperactive	Hypermetria	Follows with eyes	Follows with the l	Muscle Tone
HIPERACTIVO	MOVEXAGE	SIGUEMIRADA	MIRACABEZA	TONO
NO	NO	YES	YES	NORMAL
NO	NO	NO	NO	NORMAL
NO	NO	NO	NO	NORMAL
NO	NO	YES	YES	NORMAL
NO	NO	YES	YES	NORMAL
NO	NO	YES	YES	NORMAL
NO	NO	YES	YES	NORMAL
NO	NO	YES	YES	NORMAL
NO	NO	YES	YES	NORMAL
NO	NO	NO	NO	TENSO
NO	NO	YES	YES	NORMAL
NO	NO	YES	YES	NORMAL
NO	NO	YES	YES	TENSO
NO	NO	NO	NO	NORMAL
NO	NO	NO	NO	NORMAL
NO	NO	NO	NO	NORMAL
NO	NO	YES	YES	NORMAL
NO	NO	YES	YES	NORMAL
NO	NO	YES	YES	NORMAL
NO	NO	YES	NO	NORMAL
NO	NO	YES	YES	NORMAL
NO	NO	YES	YES	NORMAL
NO	NO	YES	YES	NORMAL
NO	NO	NO	NO	NORMAL
NO	NO	YES	YES	NORMAL
NO	NO	YES	YES	NORMAL
NO	NO	YES	YES	TENSO
NO	NO	NO	NO	NORMAL
NO	NO	YES	YES	NORMAL
NO	YES	YES	YES	NORMAL
NO	NO	YES	YES	NORMAL
YES	NO	YES	YES	NORMAL
NO	NO	NO	NO	TENSO
NO	NO	YES	YES	NORMAL
NO	NO	YES	NO	NORMAL
NO	NO	YES	YES	NORMAL
NO	NO	YES	YES	NORMAL
YES	YES	YES	NO	NORMAL
NO	NO	YES	YES	NORMAL
YES	NO	YES	YES	NORMAL
NO	NO	YES	YES	NORMAL
NO	NO	YES	YES	NORMAL
NO	NO	YES	YES	NORMAL
NO	NO	YES	YES	TENSO
NO	NO	NO	NO	TENSO
NO	NO	YES	YES	TENSO
NO	NO	NO	NO	NORMAL
NO	NO	YES	YES	NORMAL
YES	NO	YES	YES	TENSO
NO	NO	YES	YES	NORMAL
NO	NO	NO	YES	NORMAL

NO	NO	YES	YES	NORMAL
NO	NO	YES	YES	TENSO
NO	NO	YES	YES	NORMAL
NO	NO	YES	YES	TENSO
NO	NO	NO	NO	NORMAL
NO	NO	YES	YES	NORMAL
YES	NO	YES	NO	TENSO
NO	NO	YES	YES	TENSO
YES	NO	YES	YES	NORMAL
NO	NO	YES	YES	NORMAL
NO	NO	NO	NO	NORMAL
NO	NO	YES	YES	NORMAL
NO	NO	YES	YES	NORMAL
NO	NO	YES	YES	NORMAL
NO	NO	YES	YES	NORMAL
NO	NO	YES	YES	NORMAL
NO	NO	YES	YES	NORMAL
NO	NO	YES	YES	NORMAL
NO	NO	YES	YES	NORMAL
YES	NO	YES	YES	TENSO
NO	NO	NO	NO	TENSO
NO	NO	YES	YES	NORMAL
NO	NO	YES	YES	NORMAL
NO	NO	YES	YES	NORMAL
NO	NO	YES	YES	NORMAL
NO	NO	NO	NO	NORMAL
NO	NO	YES	YES	NORMAL
YES	NO	YES	YES	NORMAL
NO	NO	NO	YES	NORMAL
NO	NO	YES	YES	NORMAL
NO	NO	YES	YES	NORMAL
NO	NO	YES	YES	NORMAL
NO	NO	YES	YES	NORMAL
YES	NO	NO	NO	NORMAL
NO	NO	YES	YES	NORMAL
NO	NO	YES	YES	TENSO
YES	NO	YES	YES	NORMAL
NO	NO	YES	YES	TENSO
NO	NO	YES	YES	TENSO
YES	YES	YES	YES	NORMAL
NO	YES	YES	YES	TENSO
NO	NO	YES	YES	NORMAL
NO	NO	YES	YES	RELAJADO
NO	NO	YES	YES	NORMAL
YES	YES	YES	YES	NORMAL
NO	NO	YES	NO	RELAJADO
NO	NO	YES	NO	RELAJADO
NO	NO	YES	YES	NORMAL
NO	NO	YES	NO	RELAJADO
NO	NO	YES	YES	NORMAL
NO	NO	YES	YES	NORMAL
NO	NO	NO	NO	RELAJADO
NO	NO	NO	NO	NORMAL

NO	NO	YES	YES	RELAJADO
NO	NO	YES	NO	NORMAL
NO	NO	YES	NO	RELAJADO
NO	NO	YES	NO	RELAJADO
NO	NO	YES	YES	RELAJADO
NO	NO	YES	YES	NORMAL
NO	NO	YES	NO	RELAJADO
NO	NO	YES	YES	NORMAL
NO	NO	YES	NO	RELAJADO
NO	NO	YES	NO	NORMAL
NO	NO	YES	YES	NORMAL
NO	NO	YES	NO	NORMAL
NO	NO	NO	YES	RELAJADO
NO	NO	NO	YES	TENSO
NO	NO	NO	NO	RELAJADO
NO	NO	NO	NO	RELAJADO
NO	NO	NO	NO	RELAJADO
NO	NO	NO	NO	RELAJADO
NO	NO	NO	NO	RELAJADO
NO	NO	NO	NO	RELAJADO
NO	NO	NO	YES	RELAJADO
NO	NO	NO	NO	RELAJADO
NO	NO	YES	YES	NORMAL
NO	NO	NO	NO	RELAJADO
NO	NO	NO	NO	RELAJADO
NO	NO	YES	YES	NORMAL
NO	NO	NO	NO	RELAJADO
NO	NO	YES	NO	RELAJADO
NO	NO	YES	NO	NORMAL
NO	NO	YES	YES	NORMAL
NO	NO	NO	NO	RELAJADO
NO	NO	NO	NO	RELAJADO
NO	NO	NO	NO	RELAJADO
NO	NO	YES	YES	RELAJADO
NO	NO	YES	NO	RELAJADO
NO	NO	YES	YES	RELAJADO
NO	NO	NO	NO	RELAJADO
NO	NO	YES	YES	NORMAL
NO	NO	NO	NO	RELAJADO
NO	NO	YES	NO	RELAJADO
NO	NO	YES	YES	NORMAL
NO	NO	YES	NO	RELAJADO
NO	NO	YES	NO	RELAJADO
NO	NO	NO	NO	RELAJADO
NO	NO	YES	NO	RELAJADO
NO	NO	YES	YES	RELAJADO
NO	NO	NO	NO	RELAJADO
NO	NO	YES	NO	NORMAL
NO	NO	YES	NO	NORMAL
NO	NO	YES	NO	RELAJADO
NO	NO	NO	NO	RELAJADO
NO	NO	NO	NO	RELAJADO
NO	NO	NO	NO	RELAJADO

NO	NO	NO	NO	RELAJADO
NO	NO	NO	NO	RELAJADO
NO	NO	NO	NO	RELAJADO
NO	NO	NO	NO	RELAJADO
NO	NO	NO	NO	RELAJADO
NO	NO	NO	NO	RELAJADO
NO	NO	NO	NO	RELAJADO
NO	NO	NO	NO	RELAJADO
NO	NO	NO	NO	RELAJADO
NO	NO	NO	NO	RELAJADO
NO	NO	NO	NO	RELAJADO
NO	NO	NO	NO	RELAJADO
NO	NO	NO	NO	RELAJADO
NO	NO	YES	YES	NORMAL
NO	NO	YES	YES	NORMAL
NO	NO	NO	YES	RELAJADO
NO	NO	NO	NO	RELAJADO
NO	NO	NO	NO	RELAJADO
NO	NO	NO	NO	NORMAL
NO	NO	NO	NO	RELAJADO
NO	NO	NO	NO	RELAJADO
NO	NO	NO	NO	RELAJADO
NO	NO	NO	NO	RELAJADO
NO	NO	NO	NO	RELAJADO
NO	NO	NO	NO	RELAJADO
NO	NO	NO	NO	RELAJADO
NO	NO	NO	NO	RELAJADO
NO	NO	NO	NO	RELAJADO
NO	NO	YES	YES	NORMAL
NO	NO	NO	NO	RELAJADO
NO	NO	NO	NO	RELAJADO
NO	NO	NO	NO	RELAJADO
NO	NO	NO	NO	TENSO
NO	NO	NO	NO	RELAJADO
NO	NO	YES	YES	RELAJADO
NO	NO	YES	YES	RELAJADO
NO	NO	YES	NO	RELAJADO
NO	NO	NO	NO	RELAJADO
NO	NO	NO	NO	RELAJADO
NO	NO	NO	NO	RELAJADO
NO	NO	NO	NO	RELAJADO
NO	NO	NO	NO	RELAJADO
NO	NO	NO	NO	RELAJADO
NO	NO	NO	NO	RELAJADO
NO	NO	YES	NO	NORMAL
NO	NO	NO	NO	RELAJADO
YES	NO	NO	NO	RELAJADO
NO	NO	NO	NO	RELAJADO

Righting Reflex	Movements	change in posture	Aggresive	Flighty
REFLEJOPOSTURAL	MOVIMIENTOS	CAMBIOPOSTURA	AGRESIVO	HUIDA
YES	NORMALES	SIN	NO	NO
YES	NORMALES	SIN	NO	NO
YES	NORMALES	SIN	NO	NO
YES	NORMALES	SIN	NO	NO
YES	NORMALES	SIN	NO	NO
YES	NORMALES	SIN	NO	NO
YES	NORMALES	SIN	NO	NO
YES	NORMALES	SIN	NO	NO
YES	AUMENTADOS	SIN	NO	YES
YES	NORMALES	SIN	NO	NO
YES	NORMALES	SIN	NO	NO
YES	NORMALES	SIN	NO	YES
YES	NORMALES	SIN	NO	NO
YES	NORMALES	SIN	NO	NO
YES	NORMALES	SIN	NO	NO
YES	NORMALES	SIN	NO	NO
YES	NORMALES	SIN	NO	NO
YES	NORMALES	SIN	NO	NO
YES	NORMALES	SIN	NO	NO
YES	NORMALES	SIN	NO	NO
YES	NORMALES	SIN	NO	YES
YES	NORMALES	SIN	NO	NO
YES	NORMALES	SIN	NO	NO
YES	NORMALES	SIN	NO	NO
YES	NORMALES	SIN	NO	NO
YES	NORMALES	SIN	NO	NO
YES	NORMALES	SIN	NO	NO
YES	NORMALES	SIN	NO	NO
YES	NORMALES	SIN	NO	NO
YES	NORMALES	SIN	NO	NO
YES	NORMALES	SIN	NO	NO
YES	NORMALES	SIN	NO	NO
YES	AUMENTADOS	SIN	NO	NO
YES	NORMALES	SIN	NO	NO
YES	NORMALES	SIN	NO	NO
YES	NORMALES	SIN	NO	YES
YES	NORMALES	SIN	NO	NO
YES	NORMALES	SIN	YES	NO
YES	NORMALES	SIN	YES	NO
YES	AUMENTADOS	SIN	NO	NO
YES	NORMALES	SIN	NO	YES
YES	NORMALES	SIN	NO	NO
YES	NORMALES	SIN	NO	YES
YES	NORMALES	SIN	YES	YES
YES	NORMALES	SIN	NO	NO
YES	AUMENTADOS	SIN	NO	NO
YES	NORMALES	SIN	NO	YES
YES	NORMALES	SIN	NO	NO
YES	NORMALES	SIN	NO	YES
YES	NORMALES	SIN	NO	YES
YES	NORMALES	SIN	NO	NO

YES	NORMALES	SIN	NO	YES
YES	NORMALES	SIN	NO	YES
YES	NORMALES	SIN	NO	NO
YES	AUMENTADOS	SIN	NO	NO
YES	NORMALES	SIN	NO	NO
YES	NORMALES	SIN	NO	NO
YES	AUMENTADOS	SIN	NO	YES
YES	AUMENTADOS	SIN	NO	NO
YES	AUMENTADOS	SIN	NO	YES
YES	NORMALES	SIN	NO	NO
YES	NORMALES	SIN	NO	NO
YES	NORMALES	SIN	NO	NO
YES	NORMALES	SIN	NO	NO
YES	NORMALES	SIN	NO	NO
YES	NORMALES	SIN	NO	YES
YES	NORMALES	SIN	NO	NO
YES	NORMALES	SIN	NO	YES
YES	NORMALES	SIN	NO	YES
YES	NORMALES	SIN	NO	YES
YES	AUMENTADOS	SIN	NO	NO
YES	NORMALES	SIN	NO	NO
YES	NORMALES	SIN	NO	NO
YES	NORMALES	SIN	NO	NO
YES	NORMALES	SIN	NO	YES
YES	NORMALES	SIN	NO	NO
YES	NORMALES	SIN	NO	NO
YES	NORMALES	SIN	NO	YES
YES	NORMALES	SIN	NO	NO
YES	NORMALES	SIN	NO	NO
YES	NORMALES	SIN	NO	NO
YES	NORMALES	SIN	NO	NO
YES	NORMALES	SIN	NO	YES
YES	NORMALES	SIN	YES	YES
YES	NORMALES	SIN	NO	YES
YES	NORMALES	SIN	NO	NO
YES	NORMALES	SIN	NO	NO
YES	NORMALES	SIN	NO	NO
YES	AUMENTADOS	SIN	NO	YES
YES	NORMALES	SIN	NO	YES
YES	AUMENTADOS	SIN	NO	YES
YES	AUMENTADOS	SIN	NO	NO
YES	NORMALES	CON	NO	NO
YES	DISMINUIDOS	CON	NO	NO
YES	NORMALES	SIN	NO	NO
YES	AUMENTADOS	CON	NO	NO
YES	DISMINUIDOS	CON	NO	NO
YES	DISMINUIDOS	CON	NO	NO
YES	NORMALES	SIN	NO	NO
YES	DISMINUIDOS	SIN	NO	NO
YES	DISMINUIDOS	CON	NO	YES
YES	DISMINUIDOS	CON	NO	NO
YES	DISMINUIDOS	CON	NO	YES
YES	NORMALES	SIN	NO	NO

YES	DISMINUIDOS	CON	NO	NO
NO	DISMINUIDOS	CON	NO	NO
YES	DISMINUIDOS	CON	NO	NO
YES	DISMINUIDOS	CON	NO	NO
YES	NORMALES	CON	NO	NO
YES	NORMALES	SIN	NO	YES
YES	DISMINUIDOS	CON	NO	NO
YES	DISMINUIDOS	CON	NO	YES
YES	DISMINUIDOS	CON	NO	NO
YES	AUMENTADOS	CON	NO	NO
YES	DISMINUIDOS	CON	NO	NO
YES	NORMALES	NOCAMBIA	NO	NO
YES	NORMALES	NOCAMBIA	NO	NO
YES	NORMALES	NOCAMBIA	NO	NO
YES	DISMINUIDOS	CON	NO	NO
NO	DISMINUIDOS	CON	NO	NO
NO	DISMINUIDOS	CON	NO	NO
NO	DISMINUIDOS	CON	NO	NO
NO	DISMINUIDOS	NOCAMBIA	NO	NO
YES	DISMINUIDOS	NOCAMBIA	NO	NO
YES	DISMINUIDOS	CON	NO	NO
NO	DISMINUIDOS	NOCAMBIA	NO	NO
YES	DISMINUIDOS	CON	NO	NO
YES	NORMALES	CON	NO	NO
NO	DISMINUIDOS	CON	NO	YES
YES	NORMALES	CON	NO	NO
YES	DISMINUIDOS	NOCAMBIA	NO	NO
NO	DISMINUIDOS	NOCAMBIA	NO	NO
YES	NORMALES	CON	NO	NO
YES	DISMINUIDOS	CON	NO	NO
NO	DISMINUIDOS	CON	NO	NO
NO	DISMINUIDOS	CON	NO	NO
YES	DISMINUIDOS	CON	NO	NO
YES	DISMINUIDOS	CON	NO	NO
YES	DISMINUIDOS	CON	NO	YES
YES	DISMINUIDOS	CON	NO	NO
YES	DISMINUIDOS	NOCAMBIA	NO	NO
YES	NORMALES	NOCAMBIA	YES	NO
YES	DISMINUIDOS	CON	NO	NO
YES	DISMINUIDOS	CON	NO	YES
YES	DISMINUIDOS	CON	NO	NO
YES	DISMINUIDOS	CON	NO	NO
YES	DISMINUIDOS	CON	NO	NO
YES	DISMINUIDOS	CON	NO	NO
YES	DISMINUIDOS	CON	NO	NO
YES	DISMINUIDOS	CON	NO	YES
YES	DISMINUIDOS	CON	NO	NO
YES	DISMINUIDOS	CON	NO	NO
YES	DISMINUIDOS	CON	NO	NO
YES	DISMINUIDOS	CON	NO	NO
NO	DISMINUIDOS	NOCAMBIA	NO	NO
NO	AUSENTES	NOCAMBIA	NO	NO
NO	AUSENTES	NOCAMBIA	NO	NO

NO	AUSENTES	NOCAMBIA	NO	NO
YES	DISMINUIDOS	NOCAMBIA	NO	NO
NO	DISMINUIDOS	NOCAMBIA	NO	NO
YES	AUSENTES	NOCAMBIA	NO	NO
NO	DISMINUIDOS	NOCAMBIA	NO	NO
NO	DISMINUIDOS	NOCAMBIA	NO	NO
YES	AUSENTES	NOCAMBIA	NO	NO
YES	DISMINUIDOS	CON	NO	NO
YES	DISMINUIDOS	CON	NO	NO
NO	DISMINUIDOS	NOCAMBIA	NO	NO
NO	DISMINUIDOS	NOCAMBIA	NO	NO
NO	AUSENTES	NOCAMBIA	NO	NO
NO	AUSENTES	NOCAMBIA	NO	NO
NO	AUSENTES	NOCAMBIA	NO	NO
NO	AUSENTES	NOCAMBIA	NO	NO
NO	DISMINUIDOS	NOCAMBIA	NO	NO
YES	AUSENTES	NOCAMBIA	NO	NO
NO	AUSENTES	NOCAMBIA	NO	NO
NO	AUSENTES	NOCAMBIA	NO	NO
NO	AUSENTES	NOCAMBIA	NO	NO
NO	AUSENTES	CON	NO	NO
YES	DISMINUIDOS	NOCAMBIA	NO	NO
YES	DISMINUIDOS	NOCAMBIA	NO	YES
NO	AUSENTES	NOCAMBIA	NO	NO
NO	DISMINUIDOS	NOCAMBIA	NO	NO
YES	DISMINUIDOS	CON	NO	NO
NO	DISMINUIDOS	NOCAMBIA	NO	NO
NO	DISMINUIDOS	NOCAMBIA	NO	YES
YES	DISMINUIDOS	CON	NO	NO
NO	AUSENTES	NOCAMBIA	NO	NO
NO	DISMINUIDOS	NOCAMBIA	NO	NO
YES	AUSENTES	NOCAMBIA	NO	NO
NO	AUSENTES	NOCAMBIA	NO	NO
NO	AUSENTES	NOCAMBIA	NO	NO
NO	AUSENTES	NOCAMBIA	NO	NO
YES	AUSENTES	NOCAMBIA	NO	NO
NO	DISMINUIDOS	NOCAMBIA	NO	NO
NO	DISMINUIDOS	CON	NO	NO
NO	AUSENTES	CON	NO	NO
NO	AUSENTES	NOCAMBIA	NO	NO
NO	AUSENTES	NOCAMBIA	NO	NO
YES	DISMINUIDOS	CON	NO	NO
NO	AUSENTES	NOCAMBIA	NO	NO
YES	DISMINUIDOS	CON	NO	NO
YES	AUMENTADOS	CON	NO	NO
NO	AUSENTES	NOCAMBIA	NO	NO
NO	DISMINUIDOS	CON	NO	NO
NO	DISMINUIDOS	CON	NO	NO

DATOS

Self-harming	Defensive	Exploratory	Interactive	Voluntary movs
AUTOLESIVO	DEFENSIVO	EXPLORATORIO	INTERACTIVO	VOLUNTARIOS
NO	NO	NO	NO	YES
NO	NO	NO	NO	YES
NO	NO	NO	NO	YES
NO	NO	NO	NO	YES
NO	NO	YES	NO	YES
NO	NO	NO	YES	YES
NO	YES	NO	NO	YES
NO	NO	NO	YES	YES
NO	NO	YES	YES	YES
NO	NO	NO	NO	YES
NO	NO	NO	YES	YES
NO	NO	NO	NO	YES
NO	NO	NO	NO	YES
NO	NO	NO	YES	YES
NO	YES	NO	YES	YES
NO	NO	NO	NO	YES
NO	NO	NO	YES	YES
NO	NO	YES	YES	YES
NO	NO	NO	YES	YES
NO	NO	NO	YES	YES
NO	NO	NO	NO	YES
NO	NO	NO	YES	YES
NO	NO	NO	YES	YES
NO	NO	NO	YES	YES
NO	NO	NO	YES	YES
NO	NO	NO	YES	YES
NO	YES	NO	NO	YES
NO	NO	NO	YES	YES
NO	NO	NO	YES	YES
NO	NO	NO	NO	YES
NO	NO	NO	YES	YES
NO	NO	NO	YES	YES
NO	NO	NO	NO	YES
NO	NO	YES	YES	YES
NO	NO	NO	YES	YES
NO	NO	NO	NO	YES
NO	NO	NO	YES	YES
NO	NO	NO	NO	YES
NO	NO	NO	NO	YES
NO	NO	NO	YES	YES
NO	NO	NO	YES	YES
NO	YES	NO	NO	YES
NO	NO	NO	YES	YES
NO	NO	NO	NO	YES
NO	NO	YES	NO	YES
NO	YES	NO	YES	NO
NO	NO	NO	NO	YES
NO	NO	NO	YES	YES
NO	NO	NO	NO	YES
NO	NO	NO	NO	YES
NO	NO	YES	NO	YES

NO	NO	NO	YES	YES
NO	NO	NO	YES	YES
NO	NO	NO	YES	YES
NO	NO	NO	YES	YES
NO	NO	NO	NO	YES
NO	NO	NO	YES	YES
NO	NO	NO	NO	YES
NO	NO	NO	YES	YES
NO	NO	YES	YES	YES
NO	NO	NO	NO	YES
NO	NO	NO	NO	YES
NO	NO	NO	NO	YES
NO	NO	NO	YES	YES
NO	NO	YES	NO	YES
NO	NO	NO	NO	YES
NO	NO	NO	YES	YES
NO	NO	NO	NO	YES
NO	NO	NO	NO	YES
NO	NO	NO	NO	YES
NO	NO	NO	YES	YES
NO	NO	YES	NO	YES
NO	NO	NO	YES	YES
NO	NO	YES	YES	YES
NO	NO	NO	NO	YES
NO	NO	NO	YES	YES
NO	NO	NO	YES	YES
NO	NO	NO	NO	YES
NO	YES	NO	NO	YES
NO	NO	NO	NO	YES
NO	NO	NO	YES	YES
NO	NO	NO	YES	YES
NO	NO	NO	NO	YES
NO	NO	NO	NO	YES
NO	NO	NO	NO	YES
NO	YES	NO	NO	YES
NO	NO	NO	YES	YES
NO	YES	NO	NO	YES
NO	YES	NO	NO	YES
NO	NO	NO	NO	YES
NO	NO	NO	YES	YES
NO	NO	YES	YES	YES
NO	NO	NO	YES	YES
NO	NO	NO	NO	NO
NO	NO	NO	YES	YES
NO	YES	NO	NO	YES
NO	NO	NO	NO	NO
NO	NO	NO	NO	NO
NO	NO	YES	NO	YES
NO	YES	YES	NO	YES
NO	YES	YES	NO	NO
NO	NO	NO	YES	NO
NO	NO	NO	NO	NO
NO	YES	NO	NO	YES

NO	NO	NO	YES	NO
NO	YES	NO	NO	NO
NO	NO	NO	NO	NO
NO	YES	YES	NO	NO
NO	NO	NO	NO	NO
NO	NO	NO	YES	YES
NO	NO	NO	NO	NO
NO	NO	NO	NO	YES
NO	NO	NO	NO	NO
NO	YES	YES	YES	YES
NO	NO	NO	YES	YES
NO	NO	YES	NO	YES
NO	NO	NO	YES	YES
NO	YES	NO	NO	YES
NO	NO	NO	YES	YES
NO	NO	NO	NO	NO
NO	NO	NO	NO	NO
NO	NO	NO	NO	NO
NO	NO	NO	NO	NO
NO	NO	NO	NO	NO
NO	NO	NO	NO	NO
NO	NO	NO	NO	NO
NO	NO	YES	NO	NO
NO	NO	NO	NO	NO
NO	NO	NO	NO	NO
NO	NO	NO	YES	NO
NO	NO	NO	YES	NO
NO	NO	YES	NO	NO
NO	NO	NO	NO	NO
NO	NO	NO	NO	NO
NO	NO	NO	NO	NO
NO	NO	NO	NO	NO
NO	NO	NO	NO	NO
NO	NO	NO	NO	NO
NO	NO	NO	NO	NO
NO	NO	NO	NO	YES
NO	NO	NO	NO	YES
NO	NO	NO	NO	YES
NO	NO	NO	NO	NO
NO	NO	NO	NO	NO
NO	NO	YES	NO	NO
NO	NO	NO	YES	NO
NO	NO	NO	YES	NO
NO	NO	NO	NO	NO
NO	NO	NO	NO	NO
NO	NO	NO	NO	NO
NO	NO	YES	NO	NO
NO	NO	NO	NO	NO
NO	NO	NO	YES	NO
NO	NO	NO	YES	NO
NO	NO	NO	NO	NO
NO	NO	NO	YES	YES
NO	NO	NO	NO	NO

NO	NO	NO	NO	NO
NO	NO	NO	NO	NO
NO	NO	NO	NO	NO
NO	NO	NO	YES	NO
NO	NO	NO	YES	NO
NO	NO	NO	NO	NO
NO	NO	NO	NO	NO
NO	NO	NO	NO	NO
NO	NO	YES	NO	NO
NO	NO	NO	NO	NO
NO	NO	NO	NO	NO
NO	NO	NO	NO	NO
NO	YES	NO	YES	NO
NO	NO	NO	NO	NO
NO	NO	NO	YES	NO
NO	NO	NO	YES	NO
NO	NO	NO	NO	NO
NO	NO	NO	NO	NO
NO	NO	NO	YES	NO
NO	NO	NO	NO	NO
NO	NO	NO	NO	NO
NO	NO	NO	NO	NO
NO	NO	NO	NO	NO
NO	NO	YES	NO	NO
NO	NO	YES	NO	YES
NO	NO	NO	NO	NO
NO	NO	NO	NO	NO
NO	NO	NO	NO	NO
NO	NO	NO	NO	YES
NO	NO	NO	NO	NO
NO	NO	YES	NO	YES
NO	NO	NO	NO	YES
NO	NO	NO	NO	NO
NO	NO	NO	NO	NO
NO	NO	NO	YES	NO
NO	NO	YES	NO	NO
NO	NO	NO	NO	NO
NO	NO	NO	NO	NO
NO	NO	NO	NO	NO
NO	NO	NO	NO	NO
NO	NO	NO	NO	NO
NO	NO	NO	NO	NO
NO	YES	NO	NO	NO
NO	NO	NO	NO	NO
NO	NO	NO	NO	NO
NO	NO	YES	NO	NO
NO	NO	NO	NO	NO
NO	NO	NO	NO	NO

After stimulus	Opposition/Re	Not possible	Brisk	Eye	Eyelid
TRASESTIMULO	OPOSICION	NOPOSIBLE	BRUSCOS	OJO	PARPADO
NO	NO	NO	YES	NO	NO
NO	NO	NO	NO	YES	NO
NO	NO	YES	NO	NO	NO
NO	NO	NO	NO	NO	NO
NO	NO	NO	NO	NO	NO
NO	NO	NO	NO	NO	NO
NO	NO	NO	NO	NO	NO
NO	NO	NO	NO	NO	NO
NO	NO	NO	NO	NO	NO
NO	NO	NO	NO	NO	NO
NO	NO	NO	NO	NO	NO
NO	NO	NO	NO	NO	NO
NO	NO	NO	NO	NO	NO
NO	NO	NO	NO	NO	NO
NO	NO	NO	NO	NO	NO
NO	NO	NO	NO	NO	NO
NO	NO	NO	NO	NO	NO
NO	NO	NO	NO	NO	NO
NO	NO	NO	NO	NO	NO
NO	NO	NO	NO	NO	NO
NO	NO	NO	NO	NO	NO
NO	NO	NO	NO	NO	NO
NO	NO	NO	NO	NO	NO
NO	NO	NO	NO	NO	NO
NO	NO	NO	NO	NO	NO
NO	NO	NO	NO	NO	NO
NO	NO	NO	NO	NO	NO
NO	NO	NO	NO	NO	NO
NO	NO	NO	NO	NO	NO
NO	NO	NO	YES	NO	NO
NO	NO	NO	NO	NO	NO
NO	NO	NO	NO	NO	NO
NO	NO	NO	NO	NO	NO
NO	NO	NO	NO	NO	NO
NO	NO	NO	NO	NO	NO
NO	NO	NO	NO	NO	NO
NO	NO	NO	NO	NO	NO
NO	NO	NO	NO	NO	NO
NO	NO	NO	YES	NO	NO
NO	NO	NO	NO	NO	NO
NO	YES	NO	NO	NO	NO
NO	NO	NO	NO	NO	NO
NO	YES	NO	NO	NO	NO
NO	NO	NO	NO	NO	NO
NO	NO	NO	YES	NO	NO
NO	YES	NO	NO	NO	NO
NO	NO	NO	NO	NO	NO
NO	NO	NO	NO	NO	NO
NO	NO	NO	YES	NO	NO
NO	NO	NO	NO	NO	NO
NO	NO	NO	NO	NO	NO

NO	NO	NO	NO	NO	NO
NO	NO	NO	YES	NO	NO
NO	NO	NO	NO	NO	NO
NO	NO	NO	YES	NO	NO
NO	NO	NO	NO	NO	NO
NO	NO	NO	NO	NO	NO
NO	YES	NO	NO	NO	NO
NO	NO	NO	NO	NO	NO
NO	NO	NO	YES	NO	NO
NO	NO	NO	NO	NO	NO
NO	NO	NO	NO	NO	NO
NO	NO	NO	NO	NO	NO
NO	NO	NO	NO	NO	NO
NO	NO	NO	NO	NO	NO
NO	NO	NO	NO	NO	NO
NO	NO	NO	NO	NO	NO
NO	NO	NO	NO	NO	NO
NO	NO	NO	NO	NO	NO
NO	NO	NO	NO	NO	NO
NO	NO	NO	NO	NO	NO
NO	NO	NO	NO	NO	NO
NO	NO	NO	NO	NO	NO
NO	NO	NO	YES	NO	NO
NO	NO	NO	NO	NO	NO
NO	NO	NO	NO	NO	NO
NO	NO	NO	NO	NO	NO
NO	NO	NO	NO	NO	NO
NO	NO	NO	NO	NO	NO
NO	NO	NO	NO	NO	NO
NO	NO	NO	NO	NO	NO
NO	NO	NO	NO	NO	NO
NO	NO	NO	NO	NO	NO
NO	NO	NO	NO	NO	NO
NO	NO	NO	NO	NO	NO
NO	NO	NO	NO	NO	NO
NO	NO	NO	NO	NO	NO
NO	NO	NO	NO	NO	NO
NO	NO	NO	YES	NO	NO
NO	NO	NO	NO	NO	NO
NO	NO	NO	NO	NO	NO
NO	NO	NO	YES	NO	NO
NO	NO	NO	NO	NO	NO
YES	NO	NO	NO	NO	NO
NO	NO	NO	NO	NO	NO
NO	NO	NO	NO	NO	NO
YES	YES	NO	NO	NO	NO
YES	YES	NO	NO	NO	YES
NO	NO	NO	NO	NO	NO
NO	NO	NO	NO	NO	NO
YES	YES	NO	NO	NO	NO
NO	NO	NO	NO	NO	NO
YES	NO	NO	NO	NO	NO
NO	NO	NO	NO	NO	NO

YES	NO	NO	NO	NO	NO
YES	NO	NO	NO	NO	NO
NO	NO	NO	NO	NO	NO
NO	NO	YES	NO	NO	YES
YES	NO	NO	NO	NO	NO
NO	NO	NO	NO	NO	NO
YES	NO	NO	NO	NO	NO
NO	NO	NO	NO	NO	NO
YES	YES	NO	NO	NO	YES
YES	NO	NO	NO	NO	NO
YES	NO	NO	NO	NO	NO
NO	NO	NO	NO	YES	YES
NO	NO	NO	NO	YES	YES
NO	NO	NO	NO	YES	YES
NO	NO	NO	NO	NO	NO
YES	NO	NO	NO	NO	YES
NO	NO	NO	NO	YES	NO
NO	NO	YES	NO	YES	NO
NO	NO	NO	NO	YES	NO
YES	NO	NO	NO	YES	YES
NO	YES	NO	NO	NO	NO
YES	NO	NO	NO	YES	YES
YES	NO	NO	NO	NO	NO
YES	NO	NO	NO	YES	NO
YES	NO	NO	NO	NO	NO
YES	NO	NO	NO	NO	NO
YES	YES	NO	NO	NO	YES
NO	YES	NO	NO	NO	NO
YES	NO	NO	NO	NO	NO
YES	NO	NO	NO	NO	NO
NO	NO	YES	NO	YES	YES
YES	NO	NO	NO	NO	NO
YES	NO	NO	NO	NO	NO
YES	NO	NO	NO	NO	NO
YES	YES	NO	NO	NO	NO
YES	NO	NO	NO	NO	YES
NO	NO	NO	NO	NO	YES
NO	NO	NO	NO	NO	NO
YES	NO	NO	NO	YES	NO
NO	NO	YES	NO	NO	YES
YES	NO	NO	NO	NO	NO
YES	NO	NO	NO	NO	NO
YES	NO	NO	NO	NO	YES
YES	YES	NO	NO	NO	YES
YES	NO	NO	NO	NO	NO
YES	NO	NO	NO	NO	NO
YES	NO	NO	NO	NO	YES
YES	NO	NO	NO	NO	NO
YES	NO	NO	NO	NO	NO
YES	NO	NO	NO	NO	NO
NO	NO	NO	NO	YES	NO
NO	NO	YES	NO	NO	NO
NO	NO	YES	NO	YES	YES

NO	NO	YES	NO	YES	YES
NO	NO	YES	NO	NO	NO
YES	NO	NO	YES	NO	YES
YES	NO	NO	NO	YES	YES
NO	NO	YES	NO	NO	YES
NO	NO	YES	NO	YES	NO
NO	NO	NO	NO	YES	YES
NO	NO	YES	NO	YES	YES
YES	NO	NO	NO	NO	NO
NO	NO	YES	NO	YES	YES
NO	YES	NO	NO	NO	NO
NO	NO	YES	NO	NO	NO
NO	NO	YES	NO	NO	NO
NO	NO	YES	NO	NO	NO
NO	NO	YES	NO	NO	YES
YES	NO	NO	NO	YES	YES
NO	NO	YES	NO	NO	YES
NO	NO	YES	NO	NO	NO
NO	NO	YES	NO	YES	YES
NO	NO	NO	YES	NO	YES
NO	NO	YES	NO	YES	YES
YES	NO	NO	NO	YES	NO
YES	NO	NO	NO	YES	YES
NO	NO	YES	NO	YES	YES
NO	NO	NO	NO	YES	YES
YES	NO	NO	NO	YES	YES
NO	NO	YES	NO	YES	YES
NO	YES	NO	NO	NO	YES
NO	NO	NO	NO	NO	NO
NO	NO	YES	NO	NO	YES
NO	NO	NO	NO	YES	YES
NO	NO	NO	NO	NO	YES
NO	NO	NO	NO	NO	YES
NO	NO	YES	NO	NO	NO
NO	NO	YES	NO	YES	YES
NO	NO	YES	NO	NO	YES
NO	YES	NO	NO	NO	NO
YES	NO	NO	NO	YES	YES
YES	NO	NO	NO	YES	YES
NO	NO	YES	NO	YES	YES
NO	NO	YES	NO	YES	YES
YES	NO	NO	NO	YES	YES
NO	NO	YES	NO	YES	NO
YES	NO	NO	NO	YES	NO
NO	NO	NO	NO	NO	NO
NO	NO	YES	NO	NO	NO
YES	NO	NO	YES	NO	NO
YES	NO	NO	NO	YES	YES

DATOS

Vocalizaction	Palpebral refle:	Thrid eyelid	?	Midriasis	Hypersalivatior
VOCALIZACION	PALPEBRAL	3PARPADO	NO	MIDRIASIS	SIALORREA
NO	YES	YES	NO	NO	NO
NO	YES	NO	NO	NO	NO
NO	YES	NO	NO	NO	NO
NO	YES	NO	NO	NO	NO
NO	YES	NO	NO	NO	NO
NO	YES	NO	NO	NO	NO
NO	YES	NO	NO	NO	NO
NO	YES	NO	NO	NO	NO
NO	YES	NO	NO	NO	NO
NO	YES	NO	NO	NO	NO
NO	YES	NO	NO	NO	NO
NO	YES	NO	NO	NO	NO
NO	YES	NO	NO	NO	NO
NO	YES	NO	NO	NO	NO
NO	YES	NO	NO	NO	NO
NO	YES	NO	NO	NO	NO
NO	YES	NO	NO	NO	NO
NO	YES	NO	NO	NO	NO
NO	YES	YES	NO	NO	NO
NO	YES	NO	NO	NO	NO
NO	YES	NO	NO	NO	NO
NO	YES	NO	NO	NO	NO
NO	YES	NO	NO	NO	NO
NO	YES	NO	NO	NO	NO
NO	YES	NO	NO	NO	NO
YES	YES	NO	NO	NO	NO
NO	YES	NO	NO	NO	YES
NO	YES	NO	NO	NO	NO
NO	YES	NO	NO	NO	NO
YES	YES	YES	NO	NO	NO
NO	YES	NO	NO	NO	NO
NO	YES	NO	NO	NO	NO
YES	YES	NO	NO	NO	NO
NO	YES	NO	NO	NO	NO
NO	YES	NO	NO	NO	NO
NO	YES	NO	NO	NO	NO
NO	YES	NO	NO	NO	NO
NO	YES	NO	NO	NO	NO
NO	YES	NO	NO	NO	NO
NO	YES	NO	NO	NO	YES
YES	YES	NO	NO	NO	NO
YES	YES	NO	NO	NO	NO
NO	YES	NO	NO	NO	NO
YES	YES	NO	NO	NO	NO
NO	YES	NO	NO	NO	NO
NO	YES	NO	NO	NO	NO
NO	YES	NO	NO	NO	NO
NO	YES	NO	NO	NO	NO
NO	YES	NO	NO	NO	NO
NO	YES	NO	NO	NO	NO
NO	YES	NO	NO	NO	NO

NO	YES	NO	NO	YES	NO
NO	YES	NO	NO	NO	NO
NO	YES	NO	NO	NO	NO
NO	YES	NO	NO	NO	NO
YES	YES	NO	NO	NO	NO
NO	YES	NO	NO	NO	NO
YES	YES	NO	NO	NO	NO
YES	YES	NO	NO	NO	NO
NO	YES	NO	NO	NO	NO
NO	YES	NO	NO	NO	NO
NO	YES	NO	NO	NO	NO
NO	YES	NO	NO	NO	NO
NO	YES	NO	NO	NO	NO
NO	YES	NO	NO	NO	NO
NO	YES	NO	NO	NO	YES
NO	YES	NO	NO	NO	NO
NO	YES	NO	NO	NO	NO
NO	YES	NO	NO	NO	NO
YES	YES	NO	NO	YES	NO
NO	YES	NO	NO	NO	YES
NO	YES	NO	NO	NO	NO
NO	YES	NO	NO	NO	NO
NO	YES	NO	NO	NO	NO
NO	YES	NO	NO	NO	NO
NO	YES	NO	NO	NO	NO
NO	YES	NO	NO	NO	NO
YES	YES	NO	NO	NO	NO
NO	YES	NO	NO	NO	NO
NO	YES	NO	NO	NO	NO
NO	YES	NO	NO	NO	NO
NO	YES	NO	NO	NO	NO
NO	YES	NO	NO	NO	YES
YES	YES	NO	NO	NO	NO
NO	YES	NO	NO	NO	NO
NO	YES	NO	NO	NO	NO
YES	YES	NO	NO	NO	NO
YES	YES	YES	NO	NO	NO
YES	YES	NO	NO	NO	NO
YES	YES	YES	NO	NO	NO
NO	YES	YES	NO	NO	NO
YES	YES	NO	NO	NO	NO
NO	YES	NO	NO	YES	NO
NO	YES	NO	NO	NO	NO
NO	YES	YES	NO	NO	NO
YES	YES	NO	NO	NO	NO
NO	YES	NO	NO	NO	NO
NO	YES	NO	NO	NO	YES
NO	YES	NO	NO	NO	NO
NO	YES	NO	NO	NO	NO
NO	YES	NO	NO	NO	NO
NO	YES	NO	NO	NO	NO
NO	YES	NO	NO	NO	NO
NO	YES	NO	NO	NO	YES

YES	YES	NO	NO	NO	NO
NO	YES	NO	NO	NO	NO
NO	YES	NO	NO	NO	NO
NO	YES	NO	NO	NO	NO
NO	YES	NO	NO	NO	NO
NO	YES	NO	NO	NO	NO
NO	YES	NO	NO	NO	NO
NO	YES	NO	NO	NO	NO
NO	YES	NO	NO	NO	NO
NO	YES	YES	NO	NO	NO
NO	YES	NO	NO	NO	NO
NO	YES	NO	NO	NO	NO
NO	YES	NO	NO	NO	NO
NO	YES	NO	NO	NO	NO
NO	YES	NO	NO	NO	NO
NO	YES	NO	NO	NO	NO
NO	YES	YES	NO	NO	NO
NO	YES	YES	NO	NO	NO
NO	YES	YES	NO	NO	NO
NO	YES	NO	NO	NO	NO
NO	YES	YES	NO	NO	NO
NO	YES	YES	NO	NO	NO
NO	YES	YES	NO	NO	NO
NO	YES	NO	NO	NO	NO
NO	YES	YES	NO	NO	NO
NO	YES	NO	NO	NO	NO
NO	YES	NO	NO	NO	NO
NO	YES	NO	NO	NO	NO
NO	YES	YES	NO	NO	NO
NO	YES	YES	NO	NO	NO
NO	YES	YES	NO	NO	NO
NO	YES	NO	NO	NO	NO
NO	YES	NO	NO	NO	NO
YES	YES	NO	NO	NO	NO
NO	YES	NO	NO	NO	NO
NO	YES	NO	NO	YES	YES
NO	YES	NO	NO	NO	NO
NO	YES	NO	NO	NO	NO
NO	YES	YES	NO	NO	NO
NO	YES	NO	NO	NO	NO
NO	YES	NO	NO	NO	NO
NO	YES	NO	NO	NO	NO
NO	YES	NO	NO	NO	NO
NO	YES	NO	NO	NO	NO
NO	YES	NO	NO	NO	YES
NO	YES	NO	NO	NO	NO
NO	YES	NO	NO	NO	NO
NO	YES	NO	NO	NO	NO
NO	YES	NO	NO	NO	NO
NO	YES	NO	NO	NO	NO
NO	YES	NO	NO	NO	NO
NO	YES	NO	NO	NO	NO
NO	YES	YES	NO	NO	NO

NO	YES	NO	NO	NO	NO
YES	YES	NO	NO	YES	NO
NO	YES	NO	NO	NO	NO
NO	YES	YES	NO	NO	NO
NO	YES	NO	NO	NO	NO
NO	YES	NO	NO	NO	NO
NO	YES	NO	NO	NO	NO
NO	YES	NO	NO	NO	NO
NO	YES	NO	NO	NO	NO
NO	YES	YES	NO	NO	NO
NO	YES	YES	NO	NO	NO
NO	YES	YES	NO	NO	NO
NO	YES	YES	NO	NO	NO
NO	YES	NO	NO	NO	NO
NO	NO	YES	NO	NO	YES
NO	YES	YES	NO	YES	NO
NO	YES	NO	NO	NO	NO
NO	YES	NO	NO	YES	NO
NO	YES	YES	NO	NO	NO
NO	YES	YES	NO	NO	NO
NO	YES	YES	NO	NO	NO
NO	YES	NO	NO	NO	NO
NO	YES	NO	NO	NO	NO
NO	YES	YES	NO	NO	NO
NO	YES	NO	NO	NO	NO
NO	YES	YES	NO	NO	NO
NO	YES	YES	YES	NO	NO
NO	YES	YES	NO	NO	NO
NO	YES	NO	NO	NO	NO
NO	YES	YES	NO	NO	NO
NO	YES	NO	NO	NO	NO
NO	YES	NO	NO	NO	NO
NO	YES	YES	NO	NO	NO
NO	YES	NO	NO	NO	NO
NO	YES	YES	NO	NO	NO
NO	YES	NO	NO	NO	NO
NO	YES	NO	NO	NO	NO
NO	YES	YES	NO	NO	NO
NO	YES	YES	NO	NO	NO
NO	YES	YES	NO	NO	NO
NO	YES	YES	NO	NO	NO
NO	YES	YES	NO	NO	NO
NO	YES	YES	NO	NO	NO
NO	YES	YES	NO	NO	NO
YES	YES	YES	NO	NO	NO
NO	YES	NO	NO	NO	NO
NO	YES	NO	NO	NO	NO
NO	YES	NO	YES	NO	NO

Respiratoy rate	Panting	Menace Response	Hold limb	Pinching toe
FR	JADEO	AMENAZA	COGEREXTREMIDAD	PULPEJO
NORMAL	NO	NORMAL	YES	NORMAL
DISMINUIDA	NO	DISMINUIDO	YES	NORMAL
DISMINUIDA	NO	DISMINUIDO	YES	NORMAL
NORMAL	NO	NORMAL	YES	NORMAL
NORMAL	NO	NORMAL	YES	NORMAL
NORMAL	NO	NORMAL	YES	NORMAL
NORMAL	NO	NORMAL	YES	NORMAL
NORMAL	NO	NORMAL	YES	NORMAL
AUMENTADA	NO	NORMAL	YES	NORMAL
AUMENTADA	NO	DISMINUIDO	YES	NORMAL
NORMAL	NO	NORMAL	YES	NORMAL
AUMENTADA	YES	NORMAL	YES	NORMAL
NORMAL	NO	NORMAL	YES	NORMAL
NORMAL	NO	NORMAL	YES	NORMAL
NORMAL	NO	NORMAL	NO	NORMAL
NORMAL	NO	DISMINUIDO	YES	NORMAL
NORMAL	NO	NORMAL	NO	NORMAL
NORMAL	NO	NORMAL	YES	NORMAL
NORMAL	NO	NULO	YES	NORMAL
NORMAL	NO	NORMAL	YES	NORMAL
NORMAL	NO	NORMAL	YES	NORMAL
NORMAL	NO	NORMAL	YES	NORMAL
AUMENTADA	NO	NORMAL	YES	NORMAL
NORMAL	NO	NORMAL	YES	NORMAL
NORMAL	NO	NORMAL	YES	NORMAL
AUMENTADA	YES	NORMAL	YES	NORMAL
NORMAL	NO	NORMAL	YES	NORMAL
NORMAL	NO	NORMAL	YES	NORMAL
NORMAL	NO	NORMAL	YES	NORMAL
AUMENTADA	NO	NORMAL	YES	NORMAL
NORMAL	NO	NORMAL	YES	NORMAL
AUMENTADA	YES	NORMAL	NO	AUMENTADO
AUMENTADA	YES	AUMENTADO	NO	AUMENTADO
NORMAL	NO	NORMAL	YES	NORMAL
NORMAL	NO	NORMAL	YES	NORMAL
NORMAL	NO	NORMAL	NO	NORMAL
NORMAL	YES	NORMAL	YES	NORMAL
AUMENTADA	NO	NORMAL	YES	NORMAL
NORMAL	NO	NORMAL	NO	NORMAL
DISMINUIDA	YES	NORMAL	YES	NORMAL
NORMAL	NO	NORMAL	YES	NORMAL
AUMENTADA	YES	NORMAL	YES	NORMAL
NORMAL	NO	NORMAL	YES	NORMAL
AUMENTADA	YES	NORMAL	YES	NORMAL
AUMENTADA	YES	NORMAL	YES	NORMAL
AUMENTADA	NO	NORMAL	NO	AUMENTADO
NORMAL	NO	NORMAL	NO	NORMAL
AUMENTADA	NO	NORMAL	YES	NORMAL
AUMENTADA	NO	NORMAL	YES	NORMAL
NORMAL	NO	NORMAL	YES	NORMAL
AUMENTADA	YES	NORMAL	NO	NORMAL

NORMAL	NO	NORMAL	YES	NORMAL
AUMENTADA	YES	NORMAL	YES	NORMAL
NORMAL	NO	NORMAL	YES	NORMAL
NORMAL	NO	NORMAL	NO	NORMAL
AUMENTADA	YES	NORMAL	YES	NORMAL
NORMAL	NO	NORMAL	YES	NORMAL
AUMENTADA	YES	NORMAL	YES	NORMAL
AUMENTADA	NO	NORMAL	YES	AUMENTADO
AUMENTADA	YES	NORMAL	YES	AUMENTADO
NORMAL	NO	NORMAL	YES	NORMAL
NORMAL	NO	NORMAL	YES	NORMAL
NORMAL	NO	NORMAL	YES	NORMAL
NORMAL	NO	NORMAL	YES	NORMAL
NORMAL	NO	NORMAL	YES	NORMAL
AUMENTADA	YES	NORMAL	YES	NORMAL
NORMAL	NO	NORMAL	YES	NORMAL
NORMAL	NO	NORMAL	NO	NORMAL
NORMAL	NO	NORMAL	NO	NORMAL
NORMAL	YES	NORMAL	NO	NORMAL
AUMENTADA	NO	NORMAL	NO	NORMAL
NORMAL	NO	NORMAL	YES	NORMAL
NORMAL	NO	NORMAL	YES	NORMAL
AUMENTADA	NO	NORMAL	YES	NORMAL
AUMENTADA	YES	NORMAL	YES	NORMAL
NORMAL	NO	NORMAL	YES	NORMAL
NORMAL	NO	NORMAL	YES	NORMAL
AUMENTADA	NO	NORMAL	YES	AUMENTADO
AUMENTADA	NO	NORMAL	YES	NORMAL
NORMAL	NO	DISMINUIDO	YES	NORMAL
NORMAL	NO	NORMAL	YES	NORMAL
NORMAL	NO	NORMAL	YES	NORMAL
AUMENTADA	YES	NORMAL	YES	NORMAL
AUMENTADA	YES	NORMAL	NO	NORMAL
AUMENTADA	YES	NORMAL	YES	NORMAL
NORMAL	NO	NORMAL	YES	NORMAL
AUMENTADA	NO	NORMAL	YES	NORMAL
AUMENTADA	NO	NORMAL	NO	NORMAL
AUMENTADA	NO	AUMENTADO	YES	AUMENTADO
AUMENTADA	NO	NORMAL	YES	DISMINUIDO
AUMENTADA	NO	NORMAL	NO	NORMAL
AUMENTADA	NO	NORMAL	NO	NORMAL
AUMENTADA	YES	NORMAL	YES	DISMINUIDO
DISMINUIDA	NO	DISMINUIDO	YES	DISMINUIDO
NORMAL	NO	NORMAL	YES	NORMAL
NORMAL	NO	NORMAL	YES	NORMAL
AUMENTADA	NO	NORMAL	YES	NORMAL
AUMENTADA	YES	DISMINUIDO	YES	DISMINUIDO
NORMAL	NO	NORMAL	YES	NORMAL
AUMENTADA	YES	NORMAL	YES	NORMAL
NORMAL	NO	NORMAL	YES	NORMAL
AUMENTADA	YES	NORMAL	YES	NORMAL
DISMINUIDA	NO	DISMINUIDO	YES	DISMINUIDO
AUMENTADA	YES	NORMAL	YES	NORMAL

AUMENTADA	YES	NORMAL	YES	NORMAL
NORMAL	NO	NORMAL	YES	NORMAL
AUMENTADA	NO	DISMINUIDO	YES	NORMAL
DISMINUIDA	NO	DISMINUIDO	YES	DISMINUIDO
NORMAL	NO	NORMAL	YES	NORMAL
AUMENTADA	NO	NORMAL	YES	NORMAL
AUMENTADA	YES	NORMAL	YES	NORMAL
NORMAL	NO	NORMAL	YES	DISMINUIDO
AUMENTADA	NO	DISMINUIDO	YES	DISMINUIDO
DISMINUIDA	NO	NORMAL	YES	NORMAL
DISMINUIDA	NO	NORMAL	NO	NORMAL
NORMAL	NO	DISMINUIDO	YES	NORMAL
NORMAL	NO	NULO	YES	DISMINUIDO
DISMINUIDA	NO	DISMINUIDO	YES	DISMINUIDO
NORMAL	NO	NORMAL	YES	NORMAL
DISMINUIDA	NO	DISMINUIDO	YES	DISMINUIDO
DISMINUIDA	NO	NULO	YES	DISMINUIDO
NORMAL	NO	NULO	YES	NORMAL
DISMINUIDA	NO	DISMINUIDO	YES	DISMINUIDO
DISMINUIDA	NO	DISMINUIDO	YES	DISMINUIDO
NORMAL	NO	DISMINUIDO	YES	NULO
DISMINUIDA	NO	DISMINUIDO	YES	DISMINUIDO
NORMAL	NO	NORMAL	YES	DISMINUIDO
DISMINUIDA	NO	NULO	YES	DISMINUIDO
AUMENTADA	YES	DISMINUIDO	YES	NORMAL
NORMAL	NO	NORMAL	YES	NORMAL
DISMINUIDA	NO	DISMINUIDO	YES	NULO
DISMINUIDA	NO	DISMINUIDO	YES	DISMINUIDO
AUMENTADA	YES	NORMAL	YES	NULO
AUMENTADA	NO	NORMAL	YES	NULO
DISMINUIDA	NO	NULO	YES	DISMINUIDO
NORMAL	YES	DISMINUIDO	YES	DISMINUIDO
DISMINUIDA	YES	DISMINUIDO	YES	DISMINUIDO
NORMAL	NO	DISMINUIDO	YES	DISMINUIDO
DISMINUIDA	NO	NORMAL	YES	NORMAL
AUMENTADA	YES	DISMINUIDO	YES	DISMINUIDO
NORMAL	NO	DISMINUIDO	YES	DISMINUIDO
NORMAL	NO	NORMAL	YES	DISMINUIDO
DISMINUIDA	NO	DISMINUIDO	YES	DISMINUIDO
DISMINUIDA	NO	DISMINUIDO	YES	DISMINUIDO
NORMAL	NO	NORMAL	YES	DISMINUIDO
DISMINUIDA	NO	DISMINUIDO	YES	DISMINUIDO
NORMAL	NO	DISMINUIDO	YES	DISMINUIDO
NORMAL	NO	DISMINUIDO	YES	DISMINUIDO
DISMINUIDA	YES	DISMINUIDO	YES	DISMINUIDO
NORMAL	NO	DISMINUIDO	YES	NULO
DISMINUIDA	NO	NULO	YES	DISMINUIDO
AUMENTADA	YES	NORMAL	YES	NORMAL
NORMAL	NO	NORMAL	YES	DISMINUIDO
NORMAL	NO	DISMINUIDO	YES	NORMAL
NORMAL	NO	NULO	YES	NULO
DISMINUIDA	NO	DISMINUIDO	YES	NULO
NORMAL	NO	NULO	YES	NULO

DISMINUIDA	NO	NULO	YES	NULO
AUMENTADA	NO	DISMINUIDO	YES	DISMINUIDO
NORMAL	NO	NULO	NO	NORMAL
DISMINUIDA	NO	NORMAL	YES	NORMAL
DISMINUIDA	NO	DISMINUIDO	YES	DISMINUIDO
DISMINUIDA	NO	NULO	YES	DISMINUIDO
DISMINUIDA	NO	NULO	YES	NORMAL
DISMINUIDA	NO	NULO	YES	DISMINUIDO
NORMAL	NO	DISMINUIDO	YES	NULO
DISMINUIDA	NO	DISMINUIDO	YES	DISMINUIDO
DISMINUIDA	NO	DISMINUIDO	YES	DISMINUIDO
DISMINUIDA	NO	NULO	YES	DISMINUIDO
DISMINUIDA	NO	NULO	NO	DISMINUIDO
DISMINUIDA	NO	NORMAL	NO	DISMINUIDO
DISMINUIDA	NO	DISMINUIDO	YES	NULO
DISMINUIDA	NO	NORMAL	YES	NULO
DISMINUIDA	NO	NULO	YES	NULO
DISMINUIDA	NO	NULO	YES	NORMAL
NORMAL	NO	NORMAL	YES	NORMAL
DISMINUIDA	NO	NULO	NO	DISMINUIDO
AUMENTADA	NO	DISMINUIDO	YES	NULO
DISMINUIDA	NO	DISMINUIDO	YES	NULO
DISMINUIDA	NO	NULO	YES	NULO
DISMINUIDA	NO	NULO	YES	NULO
DISMINUIDA	NO	DISMINUIDO	YES	DISMINUIDO
DISMINUIDA	NO	NULO	YES	DISMINUIDO
NORMAL	NO	NULO	YES	NORMAL
DISMINUIDA	NO	DISMINUIDO	YES	DISMINUIDO
NORMAL	NO	DISMINUIDO	YES	NORMAL
DISMINUIDA	NO	NULO	YES	NORMAL
DISMINUIDA	NO	NORMAL	YES	DISMINUIDO
DISMINUIDA	NO	NULO	YES	NORMAL
DISMINUIDA	NO	NULO	YES	NULO
DISMINUIDA	NO	NULO	YES	NULO
DISMINUIDA	NO	NORMAL	YES	DISMINUIDO
DISMINUIDA	NO	NULO	YES	DISMINUIDO
NORMAL	NO	NORMAL	YES	NULO
NORMAL	NO	NULO	YES	NULO
NORMAL	NO	NULO	YES	NULO
DISMINUIDA	NO	NULO	YES	DISMINUIDO
DISMINUIDA	NO	NORMAL	YES	DISMINUIDO
DISMINUIDA	NO	NULO	YES	DISMINUIDO
DISMINUIDA	NO	NULO	YES	NULO
DISMINUIDA	NO	NULO	YES	NULO
DISMINUIDA	NO	NORMAL	YES	DISMINUIDO
DISMINUIDA	NO	NULO	YES	DISMINUIDO
DISMINUIDA	NO	NORMAL	YES	NULO
DISMINUIDA	NO	NULO	YES	NULO

Response to vc	Clapping	Restrain	Muzzle	Procedure
LLAMADA	PALMADA	SUJECION	BOZAL	PROCEDIMIENTO
NORMAL	NORMAL	SINSUJECION	NO	FACILMENTE
NORMAL	NORMAL	SINSUJECION	NO	IMPOSIBLE
NORMAL	DISMINUIDO	SINSUJECION	NO	FACILMENTE
NORMAL	NORMAL	SINSUJECION	NO	NO
NORMAL	NORMAL	POSIBLE	NO	FACILMENTE
NORMAL	NORMAL	POSIBLE	NO	FACILMENTE
NORMAL	NORMAL	POSIBLE	NO	FACILMENTE
NORMAL	NORMAL	POSIBLE	NO	IMPOSIBLE
NORMAL	NORMAL	POSIBLE	YES	FACILMENTE
NORMAL	NORMAL	POSIBLE	NO	IMPOSIBLE
NORMAL	NORMAL	POSIBLE	NO	IMPOSIBLE
NORMAL	NORMAL	POSIBLE	NO	IMPOSIBLE
NORMAL	NORMAL	POSIBLE	NO	IMPOSIBLE
NORMAL	NORMAL	POSIBLE	NO	IMPOSIBLE
NORMAL	NORMAL	POSIBLE	YES	IMPOSIBLE
NORMAL	NORMAL	POSIBLE	NO	FACILMENTE
NORMAL	NORMAL	POSIBLE	NO	FACILMENTE
NORMAL	NORMAL	POSIBLE	NO	FACILMENTE
NORMAL	DISMINUIDO	POSIBLE	NO	DIFICIL
NORMAL	NORMAL	POSIBLE	NO	FACILMENTE
NORMAL	NORMAL	POSIBLE	NO	FACILMENTE
NORMAL	NORMAL	POSIBLE	NO	FACILMENTE
NORMAL	NORMAL	POSIBLE	NO	IMPOSIBLE
NORMAL	NORMAL	POSIBLE	NO	IMPOSIBLE
NORMAL	NORMAL	POSIBLE	NO	FACILMENTE
NORMAL	NORMAL	POSIBLE	NO	FACILMENTE
NORMAL	NORMAL	POSIBLE	NO	FACILMENTE
NORMAL	NORMAL	POSIBLE	NO	IMPOSIBLE
NORMAL	NORMAL	POSIBLE	NO	FACILMENTE
NORMAL	NORMAL	POSIBLE	NO	IMPOSIBLE
NORMAL	NORMAL	POSIBLE	NO	FACILMENTE
NORMAL	AUMENTADO	BRUTA	YES	DIFICIL
NORMAL	AUMENTADO	BRUTA	NO	IMPOSIBLE
NORMAL	NORMAL	FIRME	NO	IMPOSIBLE
NORMAL	NORMAL	FIRME	YES	DIFICIL
NORMAL	NORMAL	FIRME	NO	IMPOSIBLE
NORMAL	NORMAL	FIRME	NO	IMPOSIBLE
NORMAL	NORMAL	FIRME	YES	DIFICIL
NORMAL	NORMAL	FIRME	YES	IMPOSIBLE
NORMAL	NORMAL	FIRME	NO	IMPOSIBLE
NORMAL	NORMAL	FIRME	NO	FACILMENTE
NORMAL	NORMAL	FIRME	NO	IMPOSIBLE
NORMAL	NORMAL	FIRME	NO	FACILMENTE
NORMAL	NORMAL	FIRME	NO	IMPOSIBLE
NORMAL	NORMAL	FIRME	NO	IMPOSIBLE
NORMAL	NORMAL	FIRME	NO	DIFICIL
NORMAL	NORMAL	FIRME	YES	FACILMENTE
NORMAL	NORMAL	FIRME	NO	IMPOSIBLE
NORMAL	NORMAL	FIRME	NO	IMPOSIBLE
NORMAL	NORMAL	FIRME	NO	FACILMENTE
NORMAL	NORMAL	FIRME	NO	IMPOSIBLE

NORMAL	NORMAL	FIRME	NO	IMPOSIBLE
NORMAL	AUMENTADO	FIRME	NO	FACILMENTE
NORMAL	NORMAL	FIRME	NO	FACILMENTE
NORMAL	NORMAL	FIRME	NO	IMPOSIBLE
NORMAL	NORMAL	FIRME	NO	FACILMENTE
NORMAL	NORMAL	FIRME	NO	FACILMENTE
NORMAL	NORMAL	FIRME	YES	IMPOSIBLE
NORMAL	NORMAL	FIRME	NO	IMPOSIBLE
NORMAL	NORMAL	FIRME	NO	FACILMENTE
NORMAL	NORMAL	FIRME	NO	FACILMENTE
NORMAL	NORMAL	FIRME	NO	IMPOSIBLE
NORMAL	NORMAL	FIRME	NO	IMPOSIBLE
NORMAL	NORMAL	FIRME	NO	IMPOSIBLE
NORMAL	NORMAL	FIRME	NO	IMPOSIBLE
NORMAL	NORMAL	FIRME	NO	DIFICIL
NORMAL	NORMAL	FIRME	NO	FACILMENTE
NORMAL	NORMAL	FIRME	YES	IMPOSIBLE
NORMAL	NORMAL	FIRME	NO	IMPOSIBLE
NORMAL	NORMAL	FIRME	NO	IMPOSIBLE
NORMAL	NORMAL	FIRME	NO	IMPOSIBLE
NORMAL	NORMAL	FIRME	NO	IMPOSIBLE
NORMAL	NORMAL	FIRME	NO	IMPOSIBLE
NORMAL	NORMAL	FIRME	NO	FACILMENTE
NORMAL	NORMAL	FIRME	YES	IMPOSIBLE
NORMAL	NORMAL	FIRME	NO	IMPOSIBLE
NORMAL	NORMAL	FIRME	NO	IMPOSIBLE
NORMAL	NORMAL	FIRME	YES	IMPOSIBLE
NORMAL	NORMAL	FIRME	NO	IMPOSIBLE
NORMAL	NORMAL	FIRME	NO	DIFICIL
NORMAL	NORMAL	FIRME	NO	IMPOSIBLE
NORMAL	NORMAL	FIRME	NO	FACILMENTE
NORMAL	NORMAL	BRUTA	NO	IMPOSIBLE
NORMAL	NORMAL	BRUTA	YES	IMPOSIBLE
NORMAL	NORMAL	BRUTA	YES	IMPOSIBLE
NORMAL	NORMAL	BRUTA	YES	IMPOSIBLE
NORMAL	NORMAL	BRUTA	NO	IMPOSIBLE
NORMAL	NORMAL	BRUTA	YES	FACILMENTE
AUMENTADO	AUMENTADO	BRUTA	NO	IMPOSIBLE
NORMAL	AUMENTADO	BRUTA	YES	IMPOSIBLE
NORMAL	NORMAL	BRUTA	NO	IMPOSIBLE
AUMENTADO	NORMAL	BRUTA	YES	IMPOSIBLE
NORMAL	NORMAL	SINSUJECION	NO	FACILMENTE
NORMAL	DISMINUIDO	SINSUJECION	NO	FACILMENTE
NORMAL	NORMAL	SINSUJECION	NO	FACILMENTE
NORMAL	NORMAL	POSIBLE	NO	DIFICIL
NORMAL	NORMAL	POSIBLE	NO	FACILMENTE
DISMINUIDO	DISMINUIDO	POSIBLE	NO	FACILMENTE
NORMAL	NORMAL	POSIBLE	NO	IMPOSIBLE
DISMINUIDO	NORMAL	POSIBLE	NO	FACILMENTE
NORMAL	NORMAL	POSIBLE	NO	FACILMENTE
NORMAL	NORMAL	POSIBLE	NO	FACILMENTE
NORMAL	DISMINUIDO	POSIBLE	YES	DIFICIL
NORMAL	NORMAL	POSIBLE	NO	FACILMENTE

NORMAL	NORMAL	POSIBLE	NO	IMPOSIBLE
NORMAL	DISMINUIDO	POSIBLE	NO	FACILMENTE
NORMAL	NORMAL	POSIBLE	NO	IMPOSIBLE
DISMINUIDO	DISMINUIDO	POSIBLE	YES	DIFICIL
DISMINUIDO	NORMAL	POSIBLE	NO	FACILMENTE
NORMAL	NORMAL	POSIBLE	YES	DIFICIL
NORMAL	DISMINUIDO	POSIBLE	NO	FACILMENTE
DISMINUIDO	DISMINUIDO	POSIBLE	NO	FACILMENTE
DISMINUIDO	NORMAL	POSIBLE	NO	DIFICIL
NORMAL	NORMAL	POSIBLE	NO	FACILMENTE
DISMINUIDO	NORMAL	POSIBLE	NO	FACILMENTE
DISMINUIDO	DISMINUIDO	SINSUJECION	NO	FACILMENTE
DISMINUIDO	DISMINUIDO	SINSUJECION	NO	FACILMENTE
DISMINUIDO	DISMINUIDO	SINSUJECION	NO	FACILMENTE
NORMAL	NORMAL	SINSUJECION	NO	FACILMENTE
DISMINUIDO	DISMINUIDO	SINSUJECION	NO	FACILMENTE
DISMINUIDO	DISMINUIDO	SINSUJECION	NO	FACILMENTE
DISMINUIDO	NULO	SINSUJECION	NO	FACILMENTE
DISMINUIDO	DISMINUIDO	SINSUJECION	NO	FACILMENTE
DISMINUIDO	DISMINUIDO	SINSUJECION	NO	FACILMENTE
NULO	NULO	SINSUJECION	NO	FACILMENTE
DISMINUIDO	DISMINUIDO	SINSUJECION	NO	FACILMENTE
DISMINUIDO	NORMAL	SINSUJECION	NO	FACILMENTE
NULO	NULO	SINSUJECION	NO	DIFICIL
DISMINUIDO	DISMINUIDO	SINSUJECION	YES	FACILMENTE
DISMINUIDO	DISMINUIDO	SINSUJECION	NO	FACILMENTE
NORMAL	DISMINUIDO	SINSUJECION	NO	FACILMENTE
NULO	DISMINUIDO	SINSUJECION	NO	FACILMENTE
DISMINUIDO	DISMINUIDO	SINSUJECION	NO	FACILMENTE
DISMINUIDO	DISMINUIDO	SINSUJECION	NO	FACILMENTE
DISMINUIDO	DISMINUIDO	SINSUJECION	NO	FACILMENTE
DISMINUIDO	DISMINUIDO	POSIBLE	NO	FACILMENTE
DISMINUIDO	DISMINUIDO	POSIBLE	NO	FACILMENTE
DISMINUIDO	NORMAL	POSIBLE	NO	FACILMENTE
DISMINUIDO	DISMINUIDO	POSIBLE	NO	FACILMENTE
DISMINUIDO	NULO	POSIBLE	NO	FACILMENTE
DISMINUIDO	DISMINUIDO	POSIBLE	NO	FACILMENTE
NORMAL	NORMAL	POSIBLE	YES	DIFICIL
DISMINUIDO	DISMINUIDO	POSIBLE	NO	FACILMENTE
DISMINUIDO	DISMINUIDO	POSIBLE	NO	FACILMENTE
DISMINUIDO	NORMAL	POSIBLE	NO	FACILMENTE
DISMINUIDO	DISMINUIDO	POSIBLE	NO	FACILMENTE
DISMINUIDO	NORMAL	POSIBLE	NO	FACILMENTE
NULO	DISMINUIDO	POSIBLE	NO	FACILMENTE
DISMINUIDO	DISMINUIDO	POSIBLE	NO	FACILMENTE
DISMINUIDO	DISMINUIDO	POSIBLE	NO	FACILMENTE
NORMAL	NORMAL	POSIBLE	NO	FACILMENTE
NULO	DISMINUIDO	POSIBLE	NO	FACILMENTE
DISMINUIDO	NULO	POSIBLE	NO	DIFICIL
DISMINUIDO	DISMINUIDO	POSIBLE	NO	IMPOSIBLE
NULO	DISMINUIDO	SINSUJECION	NO	FACILMENTE
DISMINUIDO	DISMINUIDO	SINSUJECION	NO	FACILMENTE
NULO	NORMAL	SINSUJECION	NO	FACILMENTE

NULO	NULO	SINSUJECION	NO	FACILMENTE
NULO	DISMINUIDO	SINSUJECION	NO	FACILMENTE
NULO	NULO	SINSUJECION	NO	FACILMENTE
NULO	DISMINUIDO	SINSUJECION	NO	FACILMENTE
NULO	NULO	SINSUJECION	NO	DIFICIL
NULO	NULO	SINSUJECION	NO	FACILMENTE
DISMINUIDO	DISMINUIDO	SINSUJECION	NO	FACILMENTE
DISMINUIDO	NORMAL	SINSUJECION	NO	FACILMENTE
DISMINUIDO	DISMINUIDO	SINSUJECION	NO	FACILMENTE
DISMINUIDO	DISMINUIDO	SINSUJECION	NO	FACILMENTE
DISMINUIDO	DISMINUIDO	SINSUJECION	NO	FACILMENTE
NULO	NULO	SINSUJECION	NO	FACILMENTE
DISMINUIDO	DISMINUIDO	SINSUJECION	NO	FACILMENTE
DISMINUIDO	NULO	SINSUJECION	NO	FACILMENTE
DISMINUIDO	NULO	SINSUJECION	NO	FACILMENTE
DISMINUIDO	NULO	SINSUJECION	NO	FACILMENTE
NULO	DISMINUIDO	SINSUJECION	NO	FACILMENTE
NULO	NULO	SINSUJECION	NO	FACILMENTE
NULO	NULO	SINSUJECION	NO	FACILMENTE
NULO	NULO	SINSUJECION	NO	FACILMENTE
NULO	NULO	SINSUJECION	NO	FACILMENTE
NULO	NULO	SINSUJECION	NO	FACILMENTE
NULO	DISMINUIDO	SINSUJECION	NO	FACILMENTE
NULO	NORMAL	SINSUJECION	NO	FACILMENTE
NULO	DISMINUIDO	SINSUJECION	NO	FACILMENTE
DISMINUIDO	NORMAL	SINSUJECION	NO	IMPOSIBLE
NORMAL	NORMAL	SINSUJECION	NO	FACILMENTE
NULO	DISMINUIDO	SINSUJECION	NO	FACILMENTE
NULO	NULO	SINSUJECION	NO	FACILMENTE
NULO	NULO	SINSUJECION	NO	FACILMENTE
NULO	DISMINUIDO	SINSUJECION	NO	FACILMENTE
NORMAL	NORMAL	SINSUJECION	NO	FACILMENTE
NULO	NULO	SINSUJECION	NO	FACILMENTE
NULO	NULO	SINSUJECION	NO	FACILMENTE
NULO	NULO	SINSUJECION	NO	FACILMENTE
DISMINUIDO	DISMINUIDO	SINSUJECION	NO	FACILMENTE
DISMINUIDO	DISMINUIDO	SINSUJECION	NO	FACILMENTE
NULO	NULO	SINSUJECION	NO	FACILMENTE
NULO	NULO	SINSUJECION	NO	FACILMENTE
DISMINUIDO	DISMINUIDO	SINSUJECION	NO	FACILMENTE
NULO	DISMINUIDO	SINSUJECION	NO	FACILMENTE
DISMINUIDO	DISMINUIDO	SINSUJECION	NO	FACILMENTE
NULO	NULO	SINSUJECION	NO	FACILMENTE
NULO	NULO	SINSUJECION	NO	IMPOSIBLE
NULO	DISMINUIDO	SINSUJECION	NO	NO
NULO	NULO	POSIBLE	NO	FACILMENTE
DISMINUIDO	DISMINUIDO	POSIBLE	NO	FACILMENTE
NULO	DISMINUIDO	POSIBLE	NO	FACILMENTE
