# Supplementary material for: The SIESTA (SEAAV Integrated evaluation sedation tool for anaesthesia) project: Initial development of a multifactorial sedation assessment tool for dogs
Source: PLoS One. 2020 Apr 1;15(4):e0230799. doi: 10.1371/journal.pone.0230799 (PMC7112187; doi:10.1371/journal.pone.0230799)
Supplement: S2 Data — (PDF) [file pone.0230799.s003.pdf]

Categoría	Experto	Descripción
**No sedación**	DA	Animal en pie o camina voluntariamente sin ataxia. Alerta, sigue con la mirada los movimientos del personal. Vocalización voluntaria.
	MA	Alerta. Se mueve libremente. Reacciona a estímulos con normalidad. Despierto. Atiende a su llamada.
	EB	Atento al medio que lo rodea. Mantiene la estación con normalidad. Deambulación normal. Respuesta rápida a estímulos sonoros. Respuesta rápida a estímulos táctiles
	AB	Estado mental idéntico al anterior a la administración, sea éste normal o influido por patología previa.
	CB	Alerta. Activo. Consciente. Inquieto. Intranquilo.
	MC	El paciente está igual que antes de la premedicación. Alerta y completamente reactivo a estímulos. Sigue con la mirada y con movimiento corporal normal al ir a valorar (sin contacto físico). Existe movimiento voluntario normal.
	CC	Estado mental: el animal esta alerta y consciente de lo que pasa a su alrededor. Te mira y escucha. Locomotor: capaz de coordinar todos sus movimientos (excluidos pacientes con problemas neurológicos o locomotores graves). Buen tono muscular.
	RF	El perro esta en la jaula de pie o esternal con movimientos normales. El perro esta pendiente de su alrededor, otros perros y personas. El perro atiende a la llamada y contacto (estímulos). Al contenerlo para realizarle una inyección o poner un catéter se pone nervioso y se aparta. El animal presenta una frecuencia de respiración y cardiaca entre normal a un poco elevada.
	LG	Alerta. Explora el entorno. Reactivo a cualquier estímulo
	AGdC	Alerta. Actitud normal. Energía. Estrés. Carácter
	RGV	Sin signos visibles de sedación
	PL	Alerta. En estación. Deambula. Respuesta rápida estímulos externos normales (auditivos, visuales o táctiles). Comportamiento aprensivo y reacio al manejo, lucha al sentirse inmovilizado físicamente (movimientos patas, cabecea, amenaza, gruñe, ladra, intenta morder).
	MMF	El perro está consciente, responde a estímulos verbales (por ejemplo al llamar su nombre). Camina con normalidad, el tono muscular se aprecia normal. Puede haber oposición a la sujeción manual prolongada. Hay respuesta al dolor.
	FMT	El perro está alerta, orejas se mueven con estímulos sonoros, responde a estímulos visuales, sonoro y tacto. Ojos abiertos y pupila centrada. Se pueden realizar procedimientos pero con restricción firme y más/menos bozal. Anda sin ataxia, quiere salir de la jaula cuando se abre e incita a ello, demuestra comportamiento de investigación, olisqueando todo. De pie o sentado. Puede que jadee o respire rápido cuando interactuamos con él.
	MM	Alerta. Ambulante o capaz de hacerlo. Interactivo, responde a estímulos verbales, visuales, táctiles. Expresión facial normal.
	CM	Animal en pie aparentemente no sedado. Interacciona de manera normal con el medio. Responde a todo tipo de estímulos. No se deja mover las extremidades o las aparta.
	PO	El perro está despierto y completamente alerta.
	CP	Alerta. Responde a los estímulos (auditivos, palpación). En pie. Deambula. Cabeza en posicion normal
	DP	Atento y vivaz. Atento del medio que lo rodea. Responde a estímulos táctiles y auditivos. Reacciona ante leve estimulo doloroso
	NR	Perro tranquilo o nervioso según su temperamento. Atento al entorno. Responde a estímulos auditivos y visuales (amenaza, palmada). Intenta escapar cuando se le sube a la mesa. Es necesario sujetarlo para que se quede encima de la mesa de exploración. Mueve la cabeza constantemente observando el entorno. Está de pie o sentado. Puede vocalizar intermitentemente. Musculatura tensa. Cambia de postura continuamente.
	ER	Alerta. Despierto. Responde a estímulos leves. Deambula voluntariamente. Ausencia de ataxia
	GS	Responde a estímulos externos. Interactúa con la gente y otros perros. Alerta. No se deja manipular con facilidad. Rehuye el decúbito esternal y el lateral.
	MS	Perro alerta, en movimiento, capaz de trotar (o correr, aunque es menos factible en la clínica u hospital) si se le insta a ello, que responde a órdenes e interactúa de forma intacta.
**Sedación ligera**	DA	Animal en pie con ataxia, tumbado manteniendo cabeza elevada o sentado con cabeza baja. En calma, somnoliento. Está pendiente del ambiente cercano que le rodea. Responde a estímulos externos
	MA	Somnoliento. Atontado. Reacciona a estímulos con lentitud. Atiende con lentitud. Sentado o con la cabeza agachada
	EB	Ligera disociación del medio. Mantiene la estación con normalidad. Deambulación normal. Respuesta disminuida a estímulos sonoros. Respuesta disminuida a estímulos tactiles.
	AB	Estado mental menor al anterior a la sedación. Pero mantiene estación, capacidad de caminar sin ataxia o ataxia leve y repuesta a estímulos.
	CB	Sentado. Atento. Calmado. Relajación muscular ligera. Reactivo a estímulos. Ojo posición normal, reflejo palpebral normal.
	MC	Alerta y reactividad a estímulo mantenida pero más lenta. Sigue con la mirada y el movimiento corporal lo realiza más lentamente al ir a valorarlo (sin contacto físico). Ante manipulación, el paciente se activa por completo y comportamiento es normal (anterior a sedación). Existe movimiento voluntario, aunque con frecuencia o intensidad disminuida.
	CC	Estado mental: El paciente es consciente pero ya no está alerta de lo que ocurre a su alrededor. Si era un animal de carácter agitado, su comportamiento es más calmado ahora. Aparato locomotor: Salvo enfermedad neurológica o locomotora, el paciente es capaz de coordinar sus movimientos aunque estos son más lentos.
	RF	El perro esta en la jaula de pie movimientos laterales de inestabilidad pero sigue de pie o esternal con reducción de movimientos. El perro esta menos pendiente de su alrededor, otros perros y personas. El perro atiende ligeramente menos a la llamada y contacto (estímulos), si queremos moverlo anda con lentitud. Al contenerlo para realizarle una inyección o poner un catéter se deja manipular pero tiende a apartase. El animal presenta una frecuencia de respiración y cardiaca entre normal o mas baja.

Categoría	Experto	Descripción
	LG	Permite la manipulación. Si se le deja tranquilo tiende a dormirse. Reacciona a estímulos si son moderados.
	AGdC	Ataxia. Responde a estimulación. Decúbito esternal. Calma. Tranquilidad
	RGV	Disminuye estado de alerta. Respuesta a estímulo externo acústico (palmada). Cabeza descendida ligeramente. Reflejo palpebral normal. Globo ocular en posición normal. Ligera ataxia, no se tumba o está alerta tumbado.
	PL	Alerta aunque ligeramente disminuida. En estación, deambula con lentitud. Si decúbito esternal es capaz de levantarse frente a estímulos. Respuesta más lenta estímulos externos normales (auditivos, visuales o táctiles). Comportamiento algo menos aprensivo aunque reacio al manejo (movimientos patas, cabecea, gruñe) al sentirse inmovilizado físicamente.
	MMF	Consciente pero ambula con mayor lentitud. La respuesta a estímulos sonoros es más lenta. El perro tolera sujeción manual mas prolongada, y la respuesta al dolor está disminuida. Puede caminar, pero mas lento.
	FMT	Perro sentado o en esfinge con cabeza elevada. Responde. Estímulos visuales, sonoros y tacto. Capaz de andar cuando se le saca de la jaula. Permite que se le hagan procedimientos como el catéter con restricción de un ayudante. Se ve el tercer párpado, ojos abiertos y pupila centrada. Mueve cola. Respira sin jadear.
	MM	Relajado. Responde a estímulos verbales, visuales, táctiles de forma pausada. Tendente a tumbarse y permanecer inmóvil. Capaz de caminar con normalidad o ligera ataxia.
	CM	Sentado pero que no se quiere tumbar o en pie y le tiemblan las patas. Interacciona con el medio de forma casi normal. Responde a estímulos a partir de moderado. Se deja mover las extremidades con dificultad
	PO	El perro está en decúbito esternal, adormecido pero se despierta fácilmente. El perro puede deambular.
	CP	Responde a los estímulos (auditivos, palpación). Sentado. Somnoliento. Atáxico pero puede deambular
	DP	Atento pero poco vivaz. Tiene tendencia a relajarse si se lo deja tranquilo. Atento al medio que lo rodea pero en postura relajada (por ejemplo decúbito esternal voluntario). Puede levantarse y caminar si lo desea. Reacciona ante leve estimulo doloroso (x ej posicionamiento catéter iv)
	NR	Atento al entorno, pero se muestra tranquilo. Responde a estímulos visuales y auditivos. Permanece sentado o en decúbito esternal. Es necesario sujetarlo por el collar para que no se mueva. Ojos abiertos. Mueve los ojos constantemente. No es posible llevar a cabo el procedimiento que motivó la sedación. Cambia poco de postura.
	ER	Calmado pero alerta. Responde a estímulos moderados (Por ejemplo, ruido ambiental o manipulación no dolorosa). Deambula pero necesita estimulación. Ataxia ligera. Relajado
	GS	Responde a estímulos externos (auditivos, visuales, movimiento). Interactúa menos con personas y otros perros. Rehuye el decúbito lateral y algo menos el esternal. Ligera incoordinación de movimientos. Permite la estación. Protrusión del tercer párpado.
	MS	El perro es capaz de mantenerse en estación, pero amplía su base de apoyo, o se sienta sobre el tercio posterior o adquiere posición de esfinge, pero con la cabeza en alto. No sería capaz de trotar, pero consigue andar, aun con falta de coordinación, si se le insta a ello. Alerta ante sonidos repentinos, o a que se le llame. Permitiría llevar a cabo maniobras que antes fueran más dificultosas, pero aún podría mover patas o cabeza ante maniobras simples (p. ej. poner una vía). No permitiría realizar algo doloroso (curetaje de herida).
**Sedación moderada**	DA	Animal tumbado. Pérdida de reflejo esternal. No está pendiente del ambiente que le rodea. Responde (levantar cabeza, retirada de extremidades, vocalización) a estímulos externos.
	MA	Está dormido pero reacciona a estímulos sonoros o dolorosos. Si no se le molesta se mantiene ausente. Decúbito esternal con cabeza tumbada o erguida. Dormido superficialmente. Ausencia de nerviosismo.
	EB	Animal adormecido o ausente respecto al medio. Le cuesta mantener la estación. Deambulación atáxica. Precisa de estimulación para responder a estímulos sonoros. Precisa de estimulación para responder a estímulos tactiles.
	AB	Estado mental inferior al principio a la administración, se mantiene echado y si se levanta hay ataxia evidente. Hay respuestas a estímulos con rápida vuelta al decúbito al cesar éste.
	CB	Acostado. Semi-inconsciente. Poco reactivo a estímulos. Relajación muscular moderada. Ojo semi-rotado, normal/poco reflejo palpebral
	MC	Paciente no alerta, con reactividad a estímulos disminuida. Sigue movimiento con la mirada, pero sin movimiento corporal al ir a valorarlo (sin contacto físico). Ante manipulación, el paciente no se activa de primeras salvo cuando hay molestias (y en este caso, la respuesta es más lenta y de menor intensidad).
	CC	Estado mental: El paciente está más deprimido o somnoliento. Sin estímulos, es capaz de dormirse y su capacidad de reacción ante un estímulo esta ralentizada. Aparato locomotor: ataxia evidente y relajación muscular.
	RF	El perro esta en la jaula en esternal o lateral con reducción de movimientos. El perro no esta pendiente de su alrededor, otros perros y personas. El perro para atender a estímulos de llamada o contacto hay que aumentar mucho el estimulo, para moverlo hay que empujar. Al contenerlo para realizarle una inyección o poner un catéter se deja manipular y no se aparta, puede reaccionar ligeramente con movimiento de la cabeza. El animal presenta una frecuencia de respiración y cardiaca mas baja del examen clínico inicial.
	LG	
	AGdC	Decúbito lateral. Depresión. Postrado. Ausente. Inadvertido
	RGV	Adopta decúbito lateral o esternal. Ligera respuesta a estímulo acústico (palmada). Cabeza agachada. Reflejo palpebral moderada. Ligera rotación globo ocular.
	PL	Indiferente frente a estímulos externos de intensidad moderada (auditivos, visuales o táctiles). Decúbito esternal con cabeza apoyada en suelo pero capaz de levantarla frente a estímulos externos más potentes. Si se coloca en decúbito lateral intenta/puede incorporarse a esternal. No deambula o lo hace con dificultad. No es reacio al manejo, se puede inmovilizar físicamente sin problema aunque puede reaccionar ligeramente al traccionar brazo y/o depilar.
	MMF	La respuesta a estímulos verbales como llamar su nombre está muy disminuida (solo mueve la cabeza). El perro se encuentra en decúbito y no camina sin ser estimulado. Tolera la sujeción manual en decúbito.

Categoría	Experto	Descripción
	FMT	Tercer párpado visible, ojos pueden estar cerrados o no, pero pupila centrada. No movimiento de cola. No respuesta a estímulos visuales, pero si tacto despertándose. Respuesta a estímulos auditivos despertándose. El perro permite procedimientos con cambios posturales sin sujeción o mínima, ej ecografía en dorsal, placas en lateral. El perro permite procedimiento mínimamente dolorosos ej. catéter con restricción mínima. Postura en esfinge con cabeza baja o en lateral.
	MM	Tumbado. Tendencia a dormir si no se le estimula. Capaz de levantarse con dificultad y caminar con ataxia y debilidad. Responde a estímulos visuales, verbales o táctiles vigorosos de forma lenta
	CM	Animal en decúbito esternal. Interacción con el medio claramente disminuida. Responde solo a estímulos intensos. Extremidades con leve tono muscular.
	PO	El perro esta en decúbito lateral y adormecido. El perro precisa estímulos repetidos para despertarse. El perro no puede deambular.
	CP	Tumbado. Responde a los estímulos (auditivos). Obnubilado. No ambulante. Ojo parcialmente rotado
	DP	Somnolencia y tiende a dormirse si se lo deja tranquilo y no es estimulado.Atento al medio que lo rodea solo si se lo llama o es estimulado físicamente. Decúbito (esternal o lateral) que puede modificarlo por si solo si es estimulado. Leve reacción a estimulo doloroso leve (catéter)
	NR	Perro tranquilo. Desconectado del entorno. Responde a estímulos auditivos y visuales si son fuertes. Ojos cerrados a ratos. Decúbito esternal con la cabeza entre las patas delanteras o en decúbito lateral sin sujeción. Es posible llevar a cabo el procedimiento que motiva la sedación si no requiere posiciones incómodas o es doloroso. Si se posiciona en una postura incómoda, recupera una postura más cómoda.
	ER	Calmado. Nivel de alerta reducido. Adormilado. Responde solo a estímulos fuertes (ej. ruidos fuertes o manipulación dolorosa). No deambula voluntariamente, y si lo hace presenta ataxia.
	GS	Menor respuesta a estímulos externos. No interactúa con otros perros y personas. Permite el “sentado” y el decúbito esternal. Rehuye todavía el decúbito lateral. No permite la estación. Incoordinación de movimientos. Protrusión del tercer párpado.
	MS	El perro se tumba, ya sea en decúbito esternal con la cabeza apoyada en el suelo, o en decúbito lateral. No logra mantenerse en estación, ni mantener la cabeza en alto estando tumbado. No responde a estímulos sonoros suaves, pero sí puede moverse (la cabeza, las orejas) en respuesta a un sonido repentino o fuerte (un timbre, un sonido metálico, hablar muy alto). Globo ocular en su posición normal. Reflejo palpebral intacto. No se mueve ante intervenciones sencillas y nada o poco dolorosas como poner un catéter venoso, rasurar... Responde (retirada de la zona afectada, aumento de frecuencias resp y cardiaca, movimiento de la cabeza) ante intervenciones levemente dolorosas (posible cateterización arterial, manipulación de una extremidad con una fractura, limpieza de un tumor de mama ulcerado...).
**Sedación profunda**	DA	Animal tumbado. Pérdida de reflejo esternal. No responde a estímulos externos. Puede responder a estímulos dolorosos moderado/intenso
	MA	Dormido profundamente. Sin reacción a estímulos ambientales. No reacciona a estímulos sonoros o táctiles. Sólo reacciona a estímulos dolorosos importantes. Permite sin resistencia cualquier tipo de manejo (instrumentación, etc.).
	EB	Animal dormido o estuporoso. Incapaz de mantener la estación. Incapaz de deambular. No responde a estímulos sonoros. No responde a estímulos táctiles.
	AB	El paciente se mantiene en decúbito, sin reacción a estímulos o con reacción leve que no impide la manipulación no dolorosa.
	CB	Inmóvil. Inconsciente. Arreactivo. Relajación muscular profunda. Ojo rotado, poco/sin reflejo palpebral.
	MC	Paciente no alerta, sin reactividad a estímulos. No es capaz de seguir el movimiento y no aparece actividad corporal al ir a valorarlo (sin contacto físico). Ante manipulación, el paciente no se activa ni ante procedimientos que podríamos considerar molestos.
	CC	Estado mental: el paciente esta somnoliento y no responde a estímulos exteriores. El ojo puede estar rotado medialmente aunque presenta todos los reflejos y tonos a la musculatura masetera. Aparato locomotor: el paciente no es capaz de desplazarse. A veces pueden existir espamos musculares y movimientos incoordinados de los miembros pero esporádicos y lentos.
	RF	El perro esta en la jaula en esternal (alfombra) o lateral sin movimientos. El perro esta ausente de su alrededor, otros perros y personas, no sigue con la mirada. El perro para atender a estímulos de llamada o contacto hay que aumentar mucho el estimulo, animal totalmente lacio hay que transportarlo en brazos. Ninguna contención para realizarle una inyección o poner catéter se deja manipular fácilmente y no reacciona. El animal presenta una frecuencia de respiración y cardiaca (muy) por debajo del examen clínico inicial.
	LG	Solo responde a estímulos intensos. Si se le manipula se despierta pero se duerme en cuanto lo dejamos en paz. Permanece dormido.
	AGdC	Inmovilidad. Rotación ocular ventromedial. Reflejo palpebral. Relajación muscular. Respiración profunda
	RGV	Adopta decúbito lateral o esternal. Ninguna o mínima respuesta a estímulo acústico (palmada). Cabeza agachada. Reflejo palpebral débil. Rotación ventromedial globo ocular
	PL	Completamente indiferente frente a estímulos externos de gran intensidad (auditivos, visuales o táctiles). Decúbito esternal con cabeza apoyada en suelo e incapaz de levantarla incluso frente a estímulos externos potentes. Tolera decúbito lateral y no intenta/puede incorporarse. Incapaz de deambular. No es reacio al manejo, se puede inmovilizar físicamente sin problema sin reacción al traccionar del brazo y/o depilar.
	MMF	El perro está en decúbito y no responde a estímulos verbales. Es difícil diferenciar si hay consciencia o pérdida de ella. El perro no camina aun siendo estimulado a hacerlo. El tono muscular es flácido. Tolera procedimientos dolorosos moderados.
	FMT	Perro con ojos cerrados. Ojo rotado ventralmente, casi no se ve pupila. Relajación muscular. No respuesta a estímulos visuales o tacto. No respuesta o mínima a estímulos auditivos. El perro te deja hacer procedimientos dolorosos sin sujeción.
	MM	Tumbado. Dormido si no se estimula. Respuesta lenta a estímulos muy vigorosos. Incapaz de levantarse y caminar o con extrema dificultad.
	CM	Animal en decúbito lateral. No interacciona en absoluto con el medio. No responde a ningún estímulo. Extremidades flácidas

Categoría	Experto	Descripción
	PO	El perro está dormido y no se despierta ante estímulos verbales o táctiles.
	CP	Tumbado. No responde a los estímulos (auditivos, palpación). Ojo rotado. Obnubilado. No ambulante
	DP	Dormido, pero puede ser despertado si se le provoca un estimulo físico o doloroso moderado. No reacciona al posicionamiento del catéter IV. Puede abrírsele la boca pero no puede ser intubado. No reacciona ante estimulo sonoro.
	NR	Perro tranquilo y sedado. Permanece en decúbito lateral sin sujeción. No responde a estímulos auditivos ni visuales aunque sean fuertes. Ojos cerrados. Tercer párpado prolapsado. Reflejos corneal y palpebral presentes. Musculatura relajada. Puede recuperar temporalmente la consciencia durante unos instantes si hay un estímulo doloroso. Es posible llevar a cabo el procedimiento que motiva la sedación, incluso si requiere una posición incómoda o es doloroso. Permanece en la postura en que se coloca, aunque sea incómoda.
	ER	Dormido (apariencia de sueño natural). Responde únicamente a estímulos muy fuertes (ej. manipulación muy dolorosa). No deambula. Relajación muscular. Fácil de manipular
	GS	No responde a estímulos externos (ni auditivos, ni visuales, ni movimiento). No interactúa con otros perros y personas. Permite el decúbito esternal y el decúbito lateral. No ambulante; No aguanta la estación. Rotación medial del globo ocular y reflejo palpebral positivo.
	MS	El perro permanece, tumbado, en la posición en la que se le coloque. Rotación ventromedial del globo ocular. Puede o no tener reflejo palpebral. No responde a estímulos sonoros repentinos o metálicos, o a volúmenes de voz altos. Se le puede realizar sin ningún movimiento por su parte y sin cambios en variables simples como frecuencia respiratoria o cardiaca maniobras como cateterización venosa o arterial, rasurado o preparación de extremidad fracturada, curetaje de heridas o úlceras, o realizar intervenciones leves (como exéresis de masas bajo anestesia locorregional) o mayores (como cirugía ortopédica en una extremidad empleando un bloqueo locorregional).
**Excitación**	DA	Reacciona de forma exagerada a los estímulos externos, Movimientos musculares bruscos y desproporcionados, Vocalización, Inquietud, camina sin rumbo. No responde a estímulos de calma
	MA	Vocalización. Reacción agresiva. Se mueve con cierta violencia. No atiende a órdenes. Nerviosismo.
	EB	Disforia. Vocalización. Hiperquinesia. Agresividad. Hiperreflexia.
	AB	Exacerbación de la respuesta a estímulos, mayor que la anterior a la administración. Puede que sin estímulos, el estado sea parecido a la sedación leve o moderada.
	CB	Hiperactivo. Tenso. Hiperémico. Violento. Agresivo
	MC	Paciente alerta, con reactividad aumentada a los estímulos, incluso puede presentarse agresivo. Aumento de actividad al ir a valorarlo, aún sin contacto físico, con mayor actividad corporal que en el momento de la premedicación. Movimiento incluso sin estímulos externos. Manipulación difícil por aumento de actividad corporal y reactividad aumentada
	CC	En este caso el animal puede estar consciente o no. Tener un control de su aparato locomotor o no pero se muestra agitado. Puede aullar, o incluso mostrarse agresivo. Sus movimientos son violentos, rápidos y difíciles de controlar. Respiración y frecuencia cardiaca están aumentados por lo general.
	RF	El perro esta en la jaula de pie o esternal con movimientos constantes entre normales a bruscos (puede ir de un lado a otro de la jaula o movimientos incoordinados), puede rascar la jaula para intentar salir. El perro se pone nervioso ante la presencia de otros perros y personas. El perro no atiende a la llamada y contacto (estímulos), le pone mas nervioso o se asusta e intenta escapar de la jaula. Al contenerlo para realizarle una inyección o poner un catéter se pone muy nervioso, se aparta y puede intentar morder o escapar, movimientos de cabeza laterales para evitar ser contenido, esconde las patas al cogerlas. El animal presenta una frecuencia de respiración alterada (polipnea o jadeo, salivación), mucosas bucal congestiva, los ojos a veces como desorbitados y frecuencia cardiaca elevada.
	LG	Tiende a reaccionar de manera exagerada ante estímulos suaves. No permite la manipulación. Puede autolesionarse.
	AGdC	Nervioso. Agresivo. Golpe. Vocalización. Involuntario
	RGV	Muy alerta. Salivación. Vocalización. Signos de agresividad y/o huida. Incoordinación motora. Dificulta o imposibilidad de manejarlo.
	PL	Alerta, nervioso y exhibe respuesta rápida y/o exagerada frente a estímulos externos (auditivos, visuales o táctiles) normales. Vocalización intensa. Pataleo, cabeceo y/o otros movimientos de huida rápidos, incoordinados y/o desproporcionados. Midriasis, salivación, taquicardia, taquipnea.
	MMF	El perro deambula y se resiste a la sujeción manual. Hay hiperactividad.
	FMT	Perro difícil de controlar, requiere de sujeción muy firme, a menudo de varias personas. Puede mostrar comportamiento agresivo o de defensa. Responde de forma excesiva a estímulos, especialmente táctiles. Ojos abiertos, pupilas centradas, quizás párpados más abiertos de lo normal. Perro jadea, está de pie y el contacto físico no consigue calmarlo. Tiende a estar en una esquina de la jaula e intenta huir cuando se abre la jaula.
	MM	No responde a ordenes básicas. Difícil de sujetar. Vocalización Jadeo. Hiperactivo
	CM	Muy nervioso. Responde a cualquier estímulo de manera exagerada. Vocaliza. Tiene miedo. Muerde.
	PO	El perro está inquieto. El perro responde con hiperreactividad ante estímulos.
	CP	Vocalización. Da vueltas en la jaula. Rasca el suelo. Ojo central. Nistagmo. Movimientos involuntarios
	DP	El perro esta hiperactivo. Reacciona exageradamente a estímulos sonoros o dolorosos. Ladra, llora, tiene movimientos incoordinados y parece no calmarse con la presencia humana ni con palabras.
	NR	Perro nervioso. Se mueve constantemente e intenta huir. Se golpea. Es necesario emplear la fuerza para inmovilizarlo. Puede intentar morder. Vocaliza. Aúlla. Delira. Ojos abiertos. Pupilas dilatadas. Musculatura tensa. Es imposible llevar a cabo el procedimiento.
	ER	Jadea. Llora o aúlla (vocalización). Responde de manera exagerada a estímulos. Pasea incesantemente. Se golpea.
	GS	Estación. Exagerada interactuación con otros perros y personas. No se deja manipular y responde con cierta agresividad. Respuesta exagerada a estímulos. Posible dilatación pupilar. Aumento de Frecuencia cardiaca. Jadeo y/ o taquipnea

Categoría	Experto	Descripción
	MS	El perro está alerta aunque puede mostrar relajación muscular, globo ocular en posición normal, reflejo palpebral presente, puede vocalizar, mostrarse taquipneico, realiza generalmente movimientos múltiples y rápidos con la cabeza solo o con todo el cuerpo, si se le deja solo en el suelo o en una jaula puede golpearse.
