# Supplementary material for: The SIESTA (SEAAV Integrated evaluation sedation tool for anaesthesia) project: Initial development of a multifactorial sedation assessment tool for dogs
Source: PLoS One. 2020 Apr 1;15(4):e0230799. doi: 10.1371/journal.pone.0230799 (PMC7112187; doi:10.1371/journal.pone.0230799)
Supplement: S3 Data — (PDF) [file pone.0230799.s004.pdf]

	NO SEDACIÓN
Estado mental	Alerta<br>Despierto<br>Activo<br>Consciente<br>Inquieto<br>Intranquilo<br>Nervioso<br>Pendiente del alrededor<br>Atento
Movimiento	Camina<br>Sin ataxia<br>Moverse libremente<br>Deambulación normal<br>Movimientos voluntarios<br>Movimientos coordinados<br>Movimientos normales<br>Correr o trotar<br>Energía/vivaz
Postura	De pie<br>Estación<br>Esternal<br>Sentado<br>Mueve cabeza<br>Cabeza en posición normal<br>Cambia de postura voluntaria<br>Rehuye el decubito esternal y lateral
Respuesta a estímulos	Reacción normal<br>Reactivo a estimulos<br>Reacción rápida<br>Mueve cabeza como respuesta a estimulos<br>Respuesta a estimulos leves
Respuesta a estímulos visuales	Sigue con la mirada<br>Mira<br>Respuesta rápida a estímulo visual<br>Respuesta a test de amenaza
Respuesta a estímulos auditivos	Atiende a llamada<br>Respuesta rápida a estímulo sonoro<br>Escucha<br>Mueve orejas con estímulos auditivos<br>Responde a palmada

	Responde a órdenes
Respuesta a estímulos táctiles	Respuesta rápida a estímulo táctiles<br>Respuesta a contacto
Respuesta a estimulo doloroso	Responde
Comportamiento	Investigación/olisqueando<br>Estrés<br>Interactivo
Procedimiento	Reacciona al procedimiento<br>Reacción al manejo
Respuesta a la sujeción	Se opone o aparta<br>Lucha al sentirse inmovilizado<br>Sujeción firme<br>Bozal<br>Aparta extremidades cuando se le cojen<br>Escapa al subir a la mesa
Tono muscular	Normal<br>Tenso
Reflejo deglutor	
Parámetros fisiológicos	HR y FR normal o elevada<br>Jadea
Expresión facial	Normal
Ojo	Abierto
Pupila	Centrada
Vocalización	Voluntaria<br>Intermitente
Salivación	

Mucosas	

SEDACIÓN LIGERA	SEDACIÓN MODERADA
Somnoliento<br>Calmado/tranquilo<br>Atontado<br>Ligera disociación del medio<br>Consciente<br>Atención disminuida<br>Se puede despertar fácilmente<br>Pendiente del alrededor<br>Atento	Ausente<br>Sueño ligero<br>Calmado/tranquilo<br>Somnoliento<br>Deprimido<br>Obnubilado<br>Alerta reducida<br>Desconectado del medio
Ataxia<br>Sin ataxia<br>Ataxia leve<br>Deambulación normal<br>Movimientos lentos<br>Movimientos voluntarios<br>Movimientos coordinados<br>Deambulación lenta<br>Mueve la cola<br>Inmóvil<br>Energia disminuida<br>Camina si lo desea<br>Camina si se le estimula	Ataxia<br>Deambula con ataxia<br>Movimientos disminuidos<br>Oposición al movimiento<br>No puede caminar<br>Camina si se le estimula<br>Cola inmóvil<br>Cambia de postura si se estimula
De pie<br>Esternal con cabeza elevada<br>Setando con cabeza baja<br>Sentado<br>Esternal<br>Cabeza baja<br>Cambia de postura si se estimula<br>Poco cambio	Lateral<br>Pérdida de reflejo esternal<br>Esternal con cabeza elevada<br>Esternal con cabeza baja<br>Esternal<br>Cambia de postura si se estimula<br>Cabeza baja<br>Cabeza suelo<br>Reflejo esternal positivo
Respuesta a estimulos moderados<br>Reactivo a estimulos<br>Reacción lenta	Responde<br>Poco reactivo<br>Respuesta disminuida<br>Respuesta disminuida a estímulos moderados<br>Sólo responde a estímulos repetidos
Sigue con la mirada más lentamente<br>Reacción lenta<br>Responde	Sigue con la mirada sin movimiento corporal<br>No respuesta<br>Respuesta disminuida<br>Reacción lenta
Atiende a llamada con lentitud<br>Respuesta disminuida a estímulo sonoro<br>Respuesta a palmada<br>Reacción lenta<br>Responde	Responde<br>Respuesta disminuida a estímulo sonoro<br>Respuesta disminuida a estímulos moderados<br>Respuesta muy disminuida a llamada<br>Reacción lenta

Atiende a llamada	
Respuesta disminuida a estímulo táctiles<br>Reacción lenta<br>Responde	Responde<br>Respuesta disminuida a estímulo táctiles<br>Respuesta disminuida a estímulos moderados<br>Reacción lenta
Disminuida<br>Responde a estímulo leve<br>Imposible procedimiento doloroso intenso	Responde<br>Disminuida<br>No respuesta<br>Imposible procedimiento doloroso intenso
Menos interactivo	
Permite el procedimiento con sujección<br>Reacciona al procedimiento<br>No permite llevar a cabo procedimiento	No respuesta<br>Ligera respuesta<br>No respuesta a posturas incómodas
Normal<br>Se opone o aparta con lentitud<br>Permite manipulación<br>Permite sujección prolongada<br>Permite sujetar extremidades y moverlas<br>Rehuye decúbito lateral<br>Permite ser colocado en esternal	No respuesta<br>Respuesta disminuida
Relajación ligera	Relajación moderada
Normales o disminuidas	Disminuidas
Relajada	Relajada
Posición normal<br>Tercer parpado<br>Reflejo palpebral positivo<br>Abierto<br>Mueve ojos constantemente	Rotado ligeramente<br>Tercer parpado<br>Reflejo palpebrar positivo disminuido<br>Cerrados<br>Abierto<br>Reflejo palpebral positivo<br>Posición normal
Centrada	

SEDACiÓN PROFUNDA	EXCITACIÓN
Sueño profundo<br>Estuporoso<br>Inconsciente<br>Desconectado del medio<br>Es posible despertarlo<br>Obnubilado	Nervioso<br>Disfórico<br>Alerta<br>Delirando<br>Agitado<br>Miedoso
No puede caminar<br>Inmóvil<br>Movimientos incoordinados<br>No camina incluso estimulado<br>No puede estar de pie	Movimientos bruscos<br>Movimientos desproporcionados<br>Movimientos violentos<br>Hiperkinesia<br>Movimientos espontáneos<br>Movimientos constantes<br>Ataxia<br>Deambula<br>Hiperactivo
Lateral<br>Pérdida de reflejo esternal<br>Esternal con cabeza en suelo<br>Cabeza suelo	De pie<br>Esternal
No respuesta<br>Respuesta disminuida<br>Sólo responde a estímulos intensos<br>Respuesta disminuida lenta	Respuesta exagerada<br>No atiende a órdenes<br>Hiperreflexia<br>Mueve cabeza como respuesta a estimulos<br>Respuesta rápida
No respuesta	Respuesta exagerada
No respuesta<br>Respuesta disminuida a estímulos intensos	No atiende a órdenes<br>No responde a llamada<br>Respuesta exagerada<br>No se calma con palabras

No respuesta<br>Respuesta disminuida a estímulos intensos	Respuesta exagerada<br>No se calma con contacto físico
Puede responder a estimulo intenso<br>No respuesta	Respuesta exagerada
	Agresivo<br>De huida<br>Autolesión<br>Defensivo
No respuesta<br>Respuesta disminuida	Manipulación dificil<br>Dificil<br>Respuesta exagerada<br>Olfateo continuo<br>Imposible
No respuesta	Manipulación dificil<br>Respuesta exagerada<br>Aparta extremidades cuando se le cojen
Relajación intensa<br>Tono en musculatura masetera<br>Espasmos musculares esporádicos	Tenso
Presente	
Muy disminuida<br>Respiración profunda	Aumentados<br>Jadea
Relajación intensa	
Rotado<br>Cerrados<br>Reflejo palpebrar disminuido o ausente<br>Reflejo corneal presente<br>Tercer parpado	Excesivamente abiertos<br>Abierto<br>Nistagmo<br>Reflejo palpebral positivo
Desaparece	Midriasis<br>Centrada
	Ladrar<br>Aullar<br>Intensa<br>Lloriquea
	Presente

	Congestiva
