# Supplementary material for: The SIESTA (SEAAV Integrated evaluation sedation tool for anaesthesia) project: Initial development of a multifactorial sedation assessment tool for dogs
Source: PLoS One. 2020 Apr 1;15(4):e0230799. doi: 10.1371/journal.pone.0230799 (PMC7112187; doi:10.1371/journal.pone.0230799)
Supplement: Attachment — Submitted filename: To the reviewers.docx [file pone.0230799.s006.docx]

To the reviewers:

Thank you to all the reviewers for taking the time to revise our revised manuscript. We believe in the revision process and we are certain this piece of research will become better after this process.

To Reviewer 1: Thank you for your positive comments. We have tried to review the manuscript critically and taking the reviewers’ comments on board, so we appreciate very much your positive feedback.

To Reviewer 2: Thank you for taking the time to review our work again. We can appreciate that, despite our efforts to answer your comments and justify our decisions, you have not accepted them. At this point, we can only defer to the Academic Editor and the rest of the Reviewers. As we explained in the previous revision, your comments and concerns were discussed by the authors at early stages of the study design and many of them were already discussed in the manuscript’s discussion (some of them even as limitations). Issues that you perceive as a design failure warranting rejection, we perceive them as strengths of the design. For example, the broad experience and expertise of the participant group makes this tool very clinically applicable (as it can be used by anyone regardless their level of experience), similarly the use of dichotomic variables makes the tool robust and not open to subjectivity or interpretation. We hope you would reconsider your opinion, but we also trust the revision process and its fairness if you decide not to do it.

To Reviewer 3: Thank you for reviewing our manuscript, for your positive comments and for your suggestions. We have made substantial changes to the manuscript to increase the quality of the English and they are highlighted in red. as we have changed a great. We have tried to increase the consistency of the terminology throughout the manuscript by using anaesthetists, participants, etc. We hope you consider positively the changes.

Yours faithfully.

The authors
